# Damage to the Locus Coeruleus Alters the Expression of Key Proteins in Limbic Neurodegeneration

**DOI:** 10.3390/ijms25063159

**Published:** 2024-03-09

**Authors:** Francesca Biagioni, Michela Ferrucci, Gloria Lazzeri, Mariarosaria Scioli, Alessandro Frati, Stefano Puglisi-Allegra, Francesco Fornai

**Affiliations:** 1 IRCCS, Istituto di Ricovero e Cura a Carattere Scientifico, Neuromed, 86077 Pozzili, Italy; francesca.biagioni@neuromed.it (F.B.); stabulario@neuromed.it (M.S.); alessandro.frati@neuromed.it (A.F.); stefano.puglisiallegra@neuromed.it (S.P.-A.); 2 Human Anatomy, Department of Translational Research and New Technologies in Medicine and Surgery, University of Pisa, 56126 Pisa, Italy; michela.ferrucci@unipi.it (M.F.); gloria.lazzeri@unipi.it (G.L.); 3 Neurosurgery Division, Human Neurosciences Department, Sapienza University, 00135 Rome, Italy

**Keywords:** sequestosome (p62), alpha-synuclein, p-Tau, HSP70, limbic system, piriform cortex, hippocampus, dorsal striatum

## Abstract

The present investigation was designed based on the evidence that, in neurodegenerative disorders, such as Alzheimer’s dementia (AD) and Parkinson’s disease (PD), damage to the locus coeruleus (LC) arising norepinephrine (NE) axons (LC-NE) is documented and hypothesized to foster the onset and progression of neurodegeneration within target regions. Specifically, the present experiments were designed to assess whether selective damage to LC-NE axons may alter key proteins involved in neurodegeneration within specific limbic regions, such as the hippocampus and piriform cortex, compared with the dorsal striatum. To achieve this, a loss of LC-NE axons was induced by the neurotoxin N-(2-chloroethyl)-N-ethyl-2-bromobenzylamine (DSP4) in C57 Black mice, as assessed by a loss of NE and dopamine-beta-hydroxylase within target regions. In these experimental conditions, the amount of alpha-synuclein (alpha-syn) protein levels were increased along with alpha-syn expressing neurons within the hippocampus and piriform cortex. Similar findings were obtained concerning phospho-Tau immunoblotting. In contrast, a decrease in inducible HSP70-expressing neurons and a loss of sequestosome (p62)-expressing cells, along with a loss of these proteins at immunoblotting, were reported. The present data provide further evidence to understand why a loss of LC-NE axons may foster limbic neurodegeneration in AD and limbic engagement during PD.

## 1. Introduction

The norepinephrine (NE) system is composed of several nuclei within the brainstem reticular formation [1]. Among these, the major component is represented by the nucleus *Locus Coeruleus* (LC, A6), which plays a key role in modulating neuronal activity [2,3,4,5,6], which involves the sleep–waking cycle [2], modulating exploration and anxiety [7], promoting stressful conditions [8], and regulating feeding behavior and affecting endocrine and autonomic control [9,10]. During recent decades, it became more and more evident that LC promotes protective effects during a variety of acute and chronic neurodegenerative disorders [11,12,13,14]. In particular, NE axons arising from LC (LC-NE) protect against neuronal damage in a variety of degenerative disorders [15,16,17,18,19,20,21,22,23,24,25]. This evidence encompasses acute neurological insults, such as epileptic seizures [26,27,28,29,30,31,32,33], brain ischemia [34,35,36], and chronic degenerative disorders. In fact, the protective effects of LC are mostly evident at disease onset and during the course of chronic neurodegeneration, such as experimental Parkinsonism induced in rodents [37,38,39] and monkeys [40], as well as in persons affected by Parkinson’s disease (PD) [12,41,42,43]. In addition, a strong association between the loss of LC neurons and the occurrence of degenerative dementia was indicated by pioneer findings [12,44,45,46,47,48,49,50]. Similarly, damage to LC neurons was reported during mixed, degenerative, and multi-infarct dementia [51]. These early studies emphasize that, in degenerative dementia and PD, a loss of LC-NE innervation occurs according to specific nuclear topography [12,52]. The effects of a loss of LC-NE innervation are likely to be more relevant in areas where baseline NE innervation is more abundant. This explains why, even in PD, LC loss is key to producing limbic alterations [53,54,55,56]. Therefore, in the present study, a special emphasis was dedicated to studying limbic regions involved both in AD and PD that receive the highest LC-NE innervation, consisting of allo-cortical areas, such as the hippocampus [57,58,59,60,61,62,63,64] and piriform cortex [65,66], compared with dorsal striatum, which receives scattered NE innervation [67]. Thus, the protective effects of LC on dopamine (DA) containing neurons of the *substantia nigra pars compacta* (SNpc), which is well documented, remain out of the scope of the present research study. In keeping with limbic regions, the rich NE innervation of the piriform cortex is seminal for its physiological activity. In fact, a loss of NE within the limbic system and the piriform cortex leads to a loss of olfactory perception and recognition, the re-arrangements of neural circuitries, and pathological findings, such as p-Tau deposition within neurons of the piriform cortex. This is reminiscent of what occurs in mild cognitive impairment, dementia, and during limbic involvement of PD [53,54,55,56,68,69,70,71,72,73,74,75,76,77,78,79,80]. This is key when considering that the piriform cortex is early and markedly affected in AD and PD. In fact, strong protective effects of LC within this same region along with the hippocampus are clearly documented [81,82,83,84,85,86,87]. The beneficial effects of LC extend to maintain the integrity of those neural circuitries connecting the hippocampal formation and the piriform cortex, which otherwise are early affected in these degenerative disorders, as evidenced by the aggregation of p-Tau protein in these connecting fibers [81,85]. Remarkably, p-Tau is relevant both for typical degenerative dementia and the extra-striatal involvement of brain areas in PD where the olfactory cortex and hippocampus are burdened by p-Tau accumulation, which is associated with abnormal aggregation of alpha-syn [88,89,90,91,92,93,94]. This pathology within the piriform cortex and its hippocampal connection may correlate with olfactory dysfunction and cognitive impairment in AD and PD patients. This contrasts with a lack of degeneration for intrinsic striatal neurons. This is why in the present study, changes induced by damage to LC-NE axons within the limbic cortex were compared with changes occurring within the dorsal striatum.

Since LC-NE innervation affects limbic neurodegeneration, the present study also examines two proteins that may counteract disease onset and severity and protect against limbic neuropathology. This is the case for p62 [95,96] and HSP70 [97,98,99,100,101,102,103,104,105,106].

Therefore, the present study was designed to assess whether pure damage of LC-arising NE axons induced by the neurotoxin N-(2-chloroethyl)-N-ethyl-2-bromobenzylamine (DSP4) may alter, per se, at short latency (1 week), the expression of specific proteins that play a key role in neurodegeneration. In detail, immunohistochemistry was carried out to count neurons positive for alpha-synuclein (alpha-syn), the inducible isoform of heat shock protein 70 (HSP70i), phospho-Tau (p-Tau), and sequestosome (p62) within specific brain areas following DSP4 administration. The rough amount of these proteins within the very same areas was measured by immunoblotting. In choosing brain areas, allo-cortical regions were selected based on previous evidence reported above, focusing on the hippocampus and piriform cortex. In fact, as reported in the first part of the introduction, these cortical areas undergo a massive loss of NE innervation, even at the early stages of limbic degeneration in AD and PD. The loss of LC neurons in PD is also involved in promoting neuron loss within SNpc. The present study aimed to assess DSP4-induced altered protein expression within highly NE-innervated limbic regions involved in AD and PD. Therefore, DA neurons in SNpc, which are lost in PD, were not analyzed in this context. Similarly, the dorsal striatum was investigated as a brain site where NE innervation is very poor and intrinsic neuronal loss is not reported in AD or PD. The present study was carried out by selecting a specific mouse strain C57Bl/6J, which has the highest sensitivity and specificity for the NE neurotoxin DSP4. In fact, species and strain differences exist concerning the toxicity of DSP4 [107]. For instance, rats undergo combined NE and serotonin (5-HT) toxicity with mild NE loss, whereas mice are more sensitive to NE toxicity, and C57 Black mice do not undergo any effect on the 5-HT system, which instead is partially damaged in albino Swiss–Webster mice [107]. In monkeys, DSP4 was recently confirmed to produce a significant NE depletion and cell loss within LC, although 5-HT levels were not measured [108]. The aim of the present study was to assess whether a severe and selective loss of LC-NE innervation may alter the number of key proteins, which may potentially increase baseline frailty of some specific brain areas within the limbic system. In fact, the expression of these proteins is believed to be a key factor in fostering (alpha-syn, p-Tau) or counteracting (HSP70i, p62) degenerative phenomena. The expression of the very same proteins was also assessed within the dorsal striatum, which is much less involved in LC-NE denervation.

## 2. Results

### 2.1. DSP4 Induces Specific and Severe Loss of NE Terminals within Limbic Regions, Leaving Unmodified 5-HT Levels

**Figure 1:**
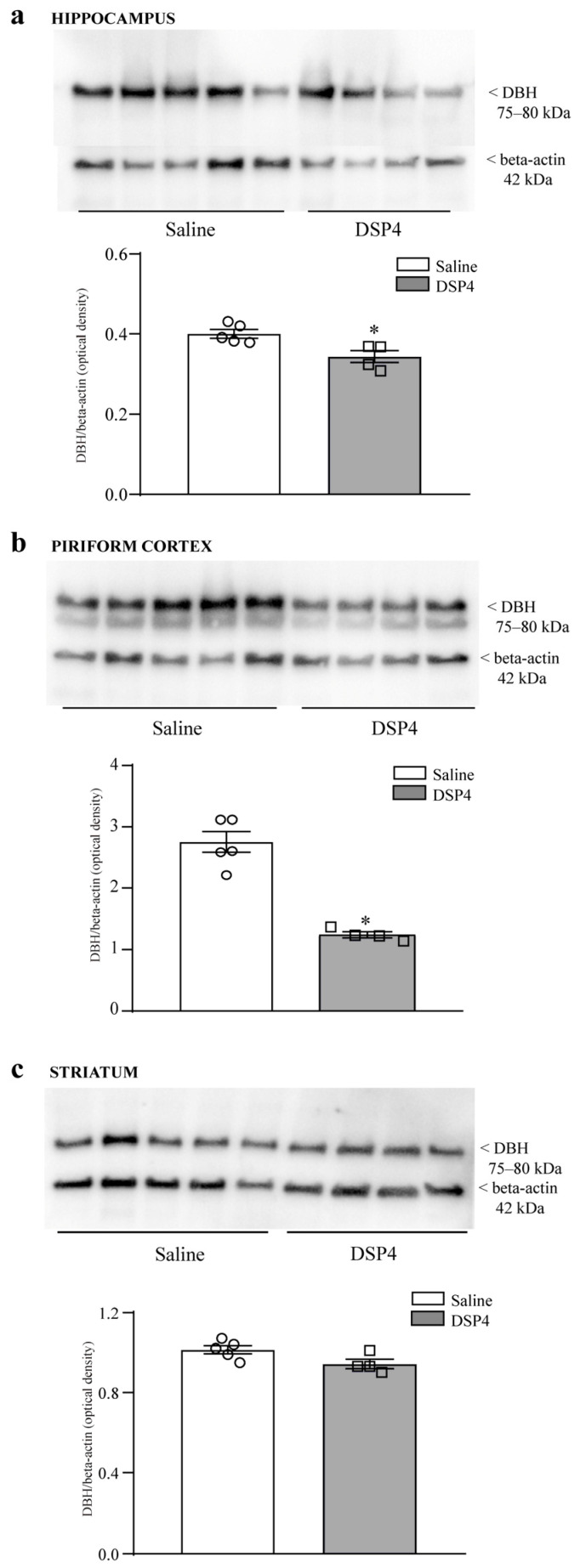
DSP4 decreases the levels of DBH within limbic regions. The figure reports immunoblotting of DBH within the hippocampus (**a**); piriform cortex (**b**); dorsal striatum (**c**). The optical density is expressed as the ratio between DBH and the housekeeping protein beta-actin within each area. A marked loss of the protein was measured within the piriform cortex and hippocampus, while a slight effect was evident in the striatum. Thus, the effects of DSP4 on DBH levels were mostly evident within limbic regions compared with the dorsal striatum. Ratios of semi-quantitative densitometry between DBH and beta-actin were provided for all areas. Values are expressed as the mean ± S.E.M. The effects obtained in the group administered saline (N = 5) and DSP4 (N = 4) were compared using ANOVA with Sheffe’s posthoc test. The null hypothesis was rejected for *p* ≤ 0.05. * *p* ≤ 0.05 compared with saline. Circles and squares represent values referred to each single saline- or DSP4—injected mouse, respectively.

The amounts of the specific enzyme for NE axon terminals, dopamine beta-hydroxylase (DBH), were assessed within the hippocampus (Figure 1a) and piriform cortex by immunoblotting (Figure 1b) at 7 days following DSP4 administration. This amount was strongly reduced in mice administered DSP4 compared with the saline-injected controls. This suggests a loss of NE-containing terminals. In contrast, the effects of DSP4 on DBH levels within the mouse striatum were negligible (Figure 1c). Thus, the loss of protein was evident within limbic regions and was the most severe within the piriform cortex, which receives the highest amount of NE innervation compared to all cortical areas. In contrast, within the dorsal striatum of DSP4-injected mice, DBH levels were similar to the controls.

**Figure 2:**
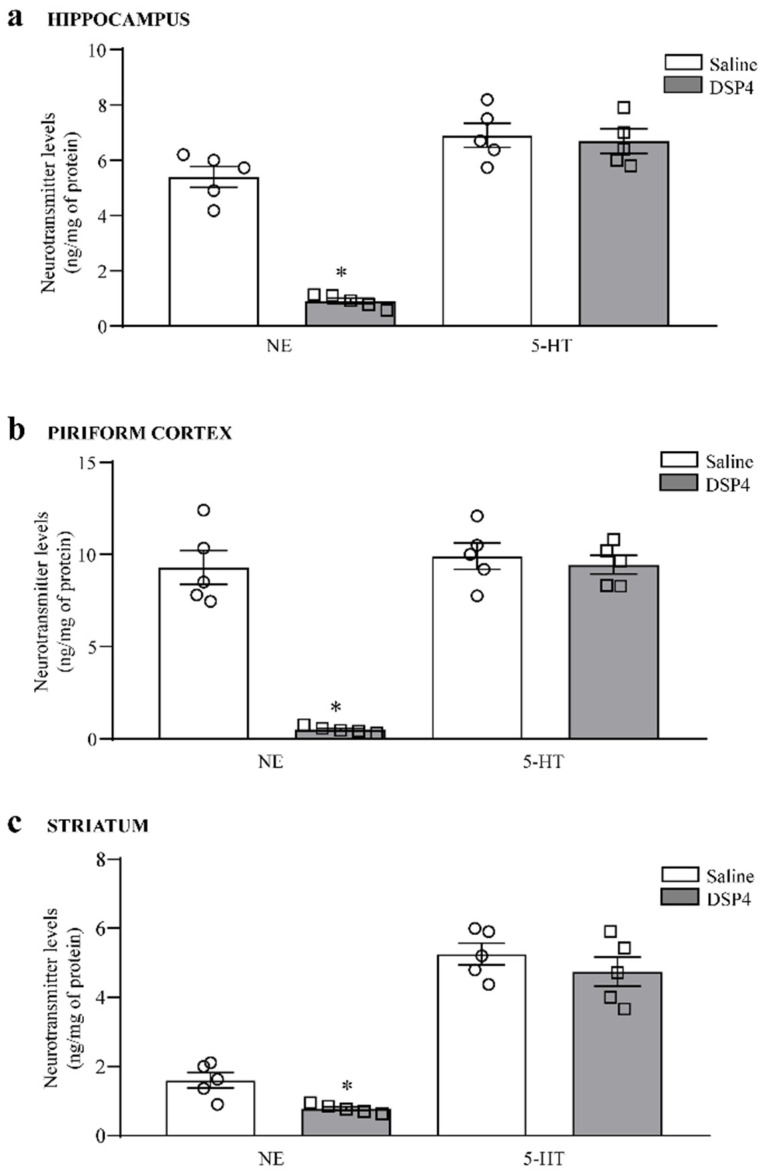
DSP-4 decreases NE levels within limbic regions and, slightly, within the dorsal striatum. Levels of NE and 5-HT were measured using HPLC-ED within the hippocampus (**a**); piriform cortex (**b**); dorsal striatum (**c**). A severe loss of NE was assessed following DSP4 in the piriform cortex and the hippocampus, while the loss of NE was moderate within the dorsal striatum. No effects were detected concerning 5-HT levels within all these areas. The number of neurotransmitters is expressed as ng/mg of tissue protein. Values are expressed as the mean ± S.E.M. The effects obtained in the group administered saline (N = 5) and DSP4 (N = 5) were compared using one-way ANOVA with Sheffè’s posthoc test. The null hypothesis was rejected for *p* ≤ 0.05. * *p* ≤ 0.05 compared with saline. Circles and squares represent values referred to each single saline- or DSP4—injected mouse, respectively.

Since optical density provides an indication, although it is not reliable, per se, to provide quantitative information concerning the amount of axon terminals within a given brain region and it cannot quantitatively establish the loss of integrity of LC-NE axon terminals, we added a quantitative neurotransmitter assay. To achieve this and validate a severe decrease in LC-NE axon terminals produced by DSP4, we measured NE levels and 5-HT levels within site-specific brain homogenates with high-performance liquid chromatography with electrochemical detection (HPLC-ED). Remarkably, within the hippocampus, DSP-4 administration reduced NE levels to roughly 25% of the controls (Figure 2a). Such a loss was even greater within the piriform cortex, where NE levels were reduced by DSP4 to less than 10% of the controls (Figure 2b); within the dorsal striatum, the decrease in NE levels was slighter (Figure 2c), which is consistent with the poor NE innervation of this brain area specifically arising from the LC. Thus, in line with the decrease in DBH levels, the piriform cortex was mostly affected by NE loss. In this area, the amount of NE measured in baseline conditions is way higher compared with other cortical regions. This confirms the rich LC-NE innervation of the piriform cortex and the highest susceptibility of this brain region to LC-NE damage induced by DSP4. As expected, in this mouse strain, the effects of DSP4 were selective for NE terminals since none of these brain areas were affected concerning 5-HT content. The effects produced by DSP4 within the dorsal striatum were mild since NE decreased moderately, reaching half of NE content, which was measured in the controls, while DBH immunoblotting was similar. This partial effect likely depends on the variety of NE nuclei converging within the striatum to convey a few NE axon terminals, most of which are mildly affected by DSP4. In fact, the deleterious effects of DSP4 in experimental parkinsonism relate to limbic neurons and damage susceptibility of DA SNpc cells, but they do not affect intrinsic striatal neurons.

### 2.2. DSP4 Induces a Dramatic Loss of HSP70-Stained Cells within Limbic Regions

**Figure 3:**
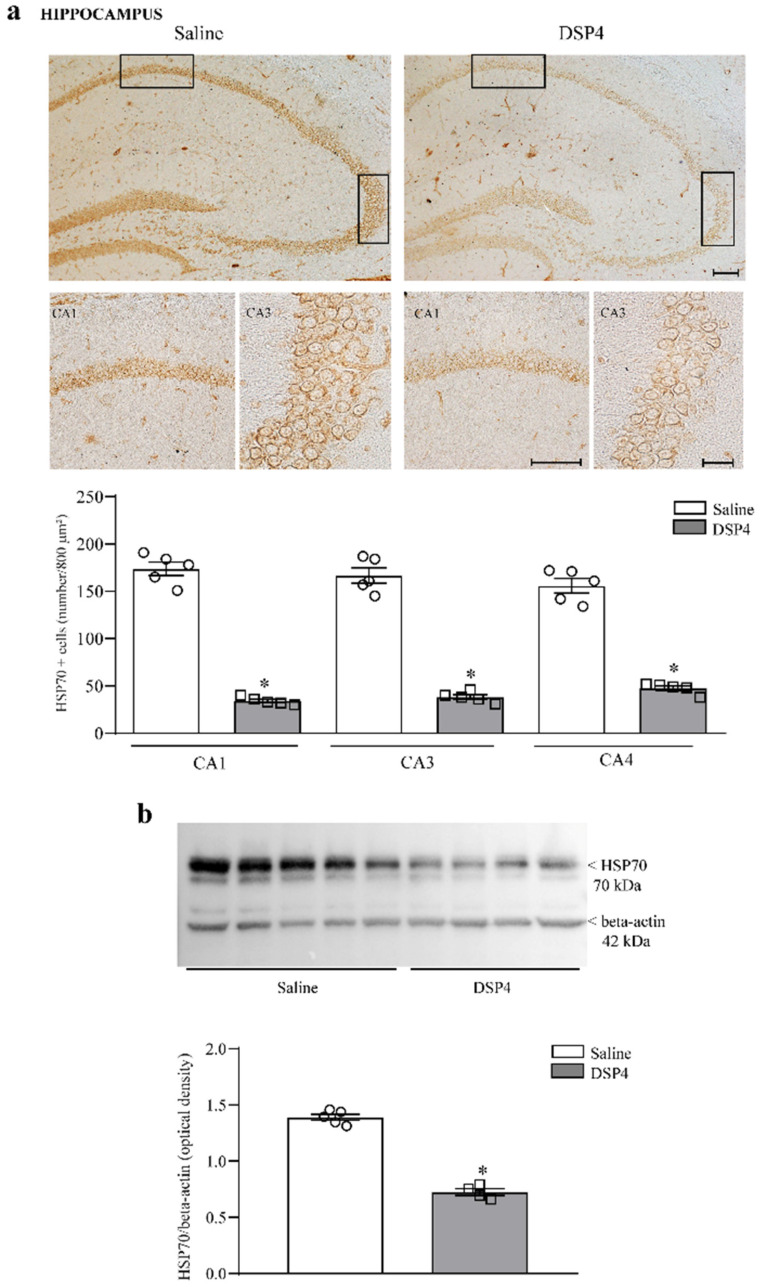
DSP4 decreases the number of HSP70i-positive neurons (cell density) and the expression of HSP70i (immunoblotting) within the hippocampus. In hippocampal slices stained for HSP70i, the density of HSP70i-positive cells is expressed as the number of cells per 800 μm^2^. When the thickness of the hippocampal folium was lower than the length of the rectangle, half of the 800 μm^2^ rectangle was used to proportionally adjust the count of neuronal density. These HSP70i-stained cells are evident in the upper part in Figure 3a, showing representative pictures where the loss of HSP70-positive neurons occurs in all (CA1, CA3, CA4) sub-fields of *cornu ammonis* in the hippocampus. The number of HSP70i-stained cells is reported in the graph, where counts of saline-injected mice are compared with DSP4-treated mice. Data are expressed as the mean ± S.E.M. In (**b**), immunoblotting for HSP70i protein is reported from the whole hippocampus. The loss of HSP70i protein is slighter compared with the loss of HSP70i-expressing cells reported in (**a**). In (**a**,**b**), the effects obtained in the groups administered either saline (N = 5) or DSP4 (N = 4) were compared using one-way ANOVA with Sheffè’s posthoc test. The null hypothesis was rejected for *p* ≤ 0.05. * *p* ≤ 0.05 compared with saline. Scale bar = 100 μm; 20 μm (insert). Circles and squares represent values referred to each single saline- or DSP4—injected mouse, respectively.

Concomitantly, with a dramatic decrease in NE innervation within the limbic regions, pre-treatment with DSP4 generates a marked loss of neuronal staining for the inducible chaperonin HSP70 within the hippocampus (Figure 3a), which occurs concomitantly with a loss of HSP70i protein, as assessed by hippocampal immunoblotting (Figure 3b). The loss of HSP70i-stained cells occurs at a similar extent within various sub-fields of *cornu ammonis* (CA, graph in Figure 3a) and it is replicated by immunoblotting the whole hippocampal region (graph in Figure 3b). When the dentate gyrus was considered for HSP70i immunostaining, a similar cell density was counted in intact and LC-NE-damaged mice, despite a slight though significant decrease in the optical density, which was measured at tissue densitometry (Supplementary Figure S1).

**Figure 4:**
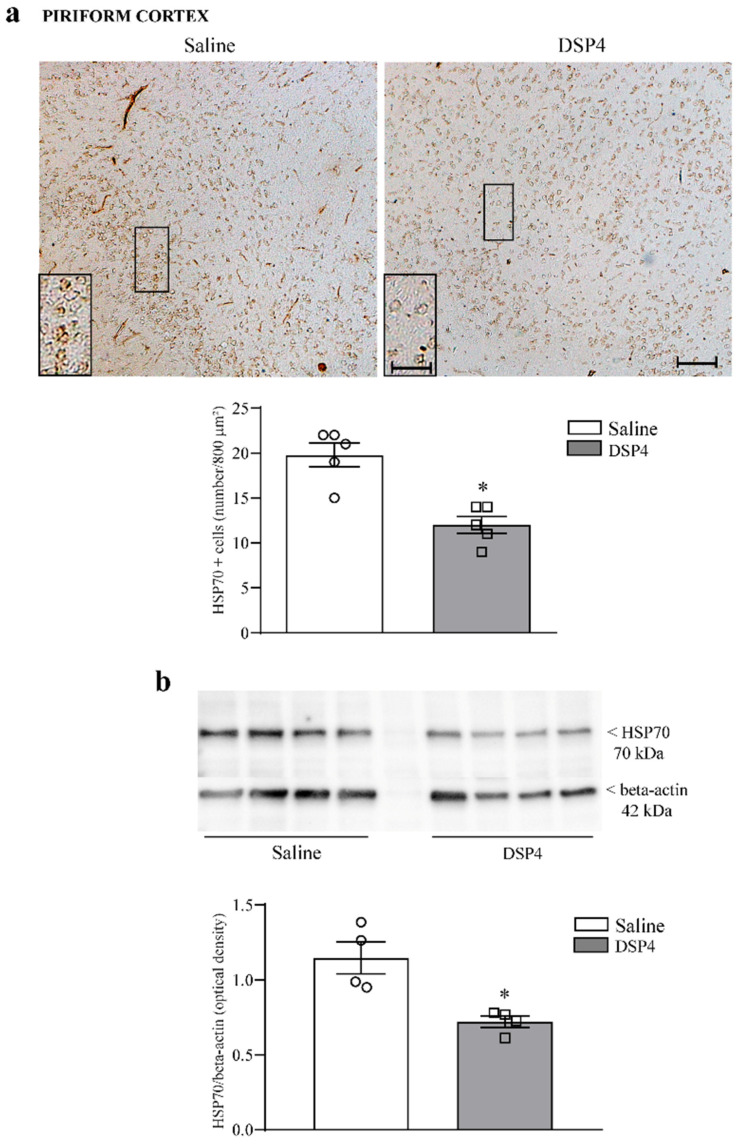
DSP4 decreases the number of HSP70i-immunopositive neurons and the expression of HSP70i protein within the piriform cortex. In slices from the anterior piriform cortex stained for HSP70i, the density of HSP70i-positive cells was expressed as the number of cells per 800 μm^2^. These cells are evident in the upper part in Figure 4a showing representative pictures, where a loss of HSP70i-positive neurons appears to occur mostly in the deep layer of the piriform cortex. This is emphasized in the insert, reporting an area at higher magnification. The number of HSP70-stained cells is reported in the graph (**a**), where counts of saline-injected mice are compared with DSP4-treated mice. Data are expressed as the mean ± S.E.M. In (**b**), immunoblotting for HSP70i protein is reported from the whole piriform cortex. In this case, the loss of HSP70i protein is slighter compared with the loss of HSP70i-expressing cells, which was previously reported within the hippocampus (Figure 3). In (**a**,**b**), the effects obtained in the groups administered either saline (N = 4) or DSP4 (N = 4) were compared using one-way ANOVA with Sheffè’s posthoc test. The null hypothesis was rejected for *p* ≤ 0.05. * *p* ≤ 0.05 compared with saline. Scale bar = 100 μm; 20 μm (insert). Circles and squares represent values referred to each single saline- or DSP4—injected mouse, respectively.

**Figure 5:**
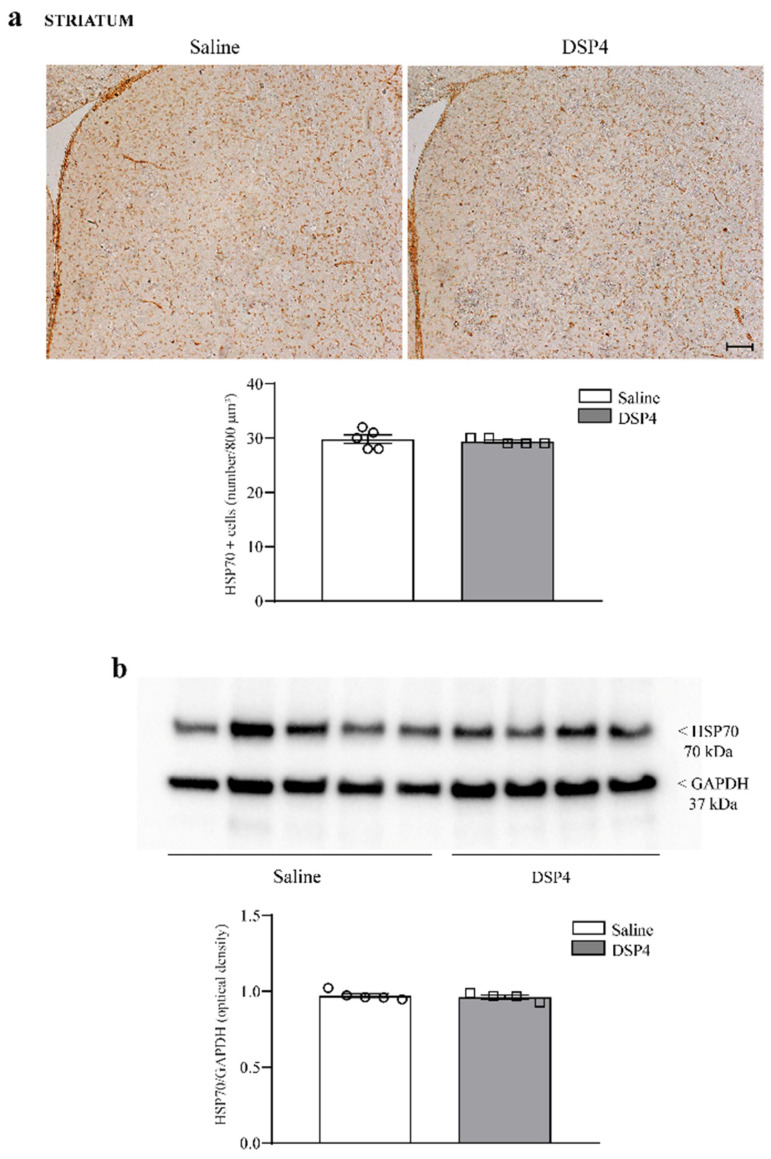
DSP4 does not modify the number of HSP70i-positive neurons and the expression of HSP70i protein within the dorsal striatum. In slices from the dorsal striatum stained for HSP70i, the density of HSP70i-stained tissue was expressed as immunopositive cells per 800 μm^2^. This cell density is evident in the upper part of the figure, showing representative pictures, where the number of HSP70i-positive cells is similar in saline- and DSP4-injected mice. This is reported in the graph, where counts of saline-injected mice are compared with DSP4-treated mice. Data are expressed as the mean ± S.E.M. In (**b**), immunoblotting for HSP70 protein within the dorsal striatum is reported. Again, no significant effects were induced by DSP4. In (**a**,**b**), the effects obtained in the groups administered saline (N = 5) and DSP4 (N = 4) were compared using one-way ANOVA with Sheffè’s posthoc test. The null hypothesis was rejected for *p* ≤ 0.05. Scale bar = 100 μm. Circles and squares represent values referred to each single saline- or DSP4—injected mouse, respectively.

The effects measured following immunoblotting (Figure 3b) were less prominent compared with results expressed by cell counts (Figure 3a). Such a difference is consistent with discrepancies between the two experimental techniques. In addition, the difference is likely to depend on the heterogeneous tissue structure selected for immunoblotting compared with the specificity of the area counted for cell density. In fact, cell count was carried out specifically within the cornu ammonis, while the immunoblotting of homogenates from wide hippocampal areas contains heterogeneous gray and white matter, leading to data dilution. Similarly, in the anterior extent of the piriform cortex, the loss of HSP70i-positive neurons was dramatic, mostly within the deep layer of this allo-cortex (Figure 4a). When immunoblotting the piriform cortex, the amount of HSP70i protein was significantly reduced in DSP4-treated mice compared with the controls (Figure 4b). In contrast, no significant effect was documented for HSP70i-immunostained neurons and HSP70i immunoblotting at the striatal level (Figure 5). It is remarkable, and somehow unexpected that, in the case of HSP70i, the decrease was less pronounced compared with the hippocampus, although the loss of NE was in excess within the piriform cortex compared with hippocampal tissue. It is likely that neuronal heterogeneity within the piriform cortex produces a variation between neurons in the sensitivity of LC-NE axons to DSP4. Such a phenomenon does not occur within homogeneous pyramidal cells occurring in the cornu ammonis, see Figure 3, or within homogeneous granule cells of dentate gyrus (Supplementary Figure S1).

### 2.3. DSP4 Elevates Alpha-Syn within Limbic Regions

**Figure 6:**
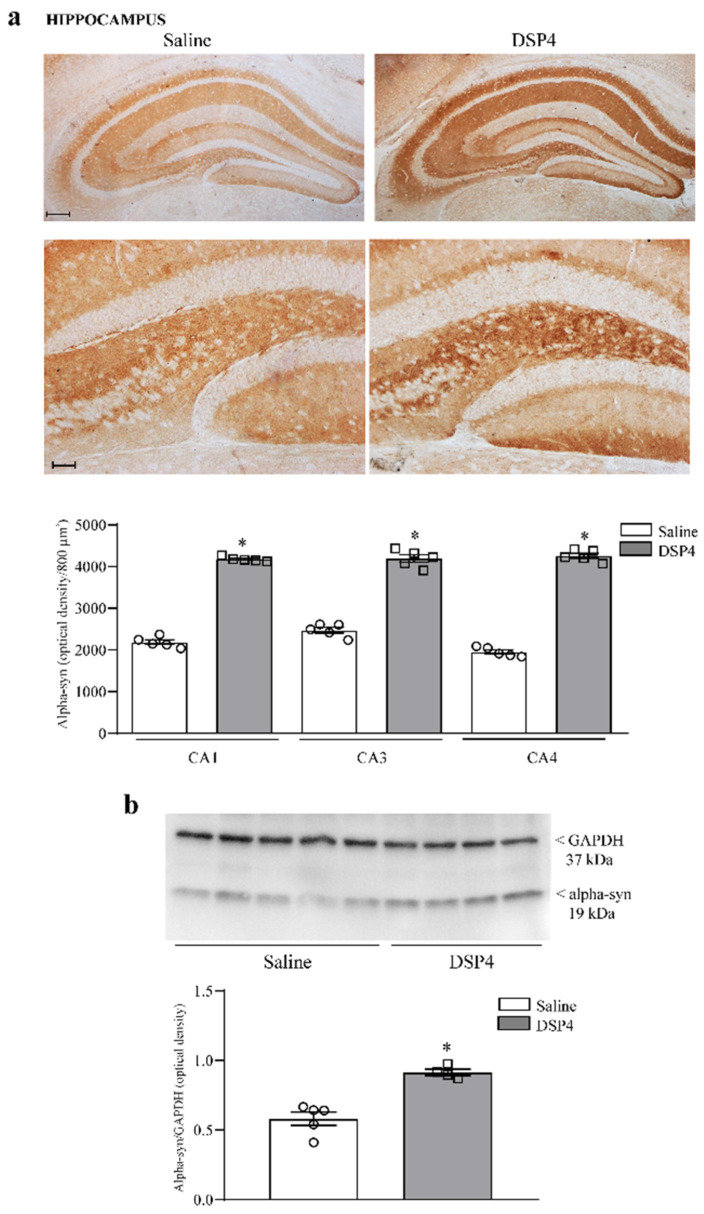
DSP4 increases the amount of alpha-syn within the hippocampus. In hippocampal slices stained for alpha-syn, the density of alpha-syn was expressed as optical tissue density since single cells were not detectable. Representative pictures are evident in the upper part in Figure 6a, showing an increase in alpha-syn in all (CA1, CA3, CA4) sub-fields of *cornu ammonis*, which extends to a lesser extent to dentate gyrus (see also Supplementary Figure S1). The amount of optical density is reported in the graph, where counts of saline-injected mice are compared with DSP4-treated mice, and all measurements were carried out considering the corpus callosum as a reference (refer to Supplementary Figure S2 for representative similar negligible staining for alpha-syn from saline-injected and DSP4-injected mice, where densitometry does not significantly differ). Data are expressed as the mean ± S.E.M. In (**b**), immunoblotting for alpha-syn protein is reported from the whole hippocampus. The increase in alpha-syn protein is comparable with the increase in alpha-syn density from hippocampal slices reported in (**a**). In (**a**,**b**), the effects obtained in the group administered saline (N = 5) and DSP4 (N = 4) were compared using one-way ANOVA with Sheffè’s posthoc test. The null hypothesis was rejected for *p* ≤ 0.05. * *p* ≤ 0.05 compared with saline. Scale bar = 100 μm; 50 μm (low and high magnification, respectively). Circles and squares represent values referred to each single saline- or DSP4—injected mouse, respectively.

Consistently, with a neuroprotective effect of LC-NE axon terminals within the forebrain, and mostly within limbic regions, the loss of LC-NE axons concomitantly leads to a dramatic increase in the detrimental protein alpha-syn at densitometry within hippocampal slices (Figure 6a) and alpha-syn protein within hippocampal immunoblotting (Figure 6b).

**Figure 7:**
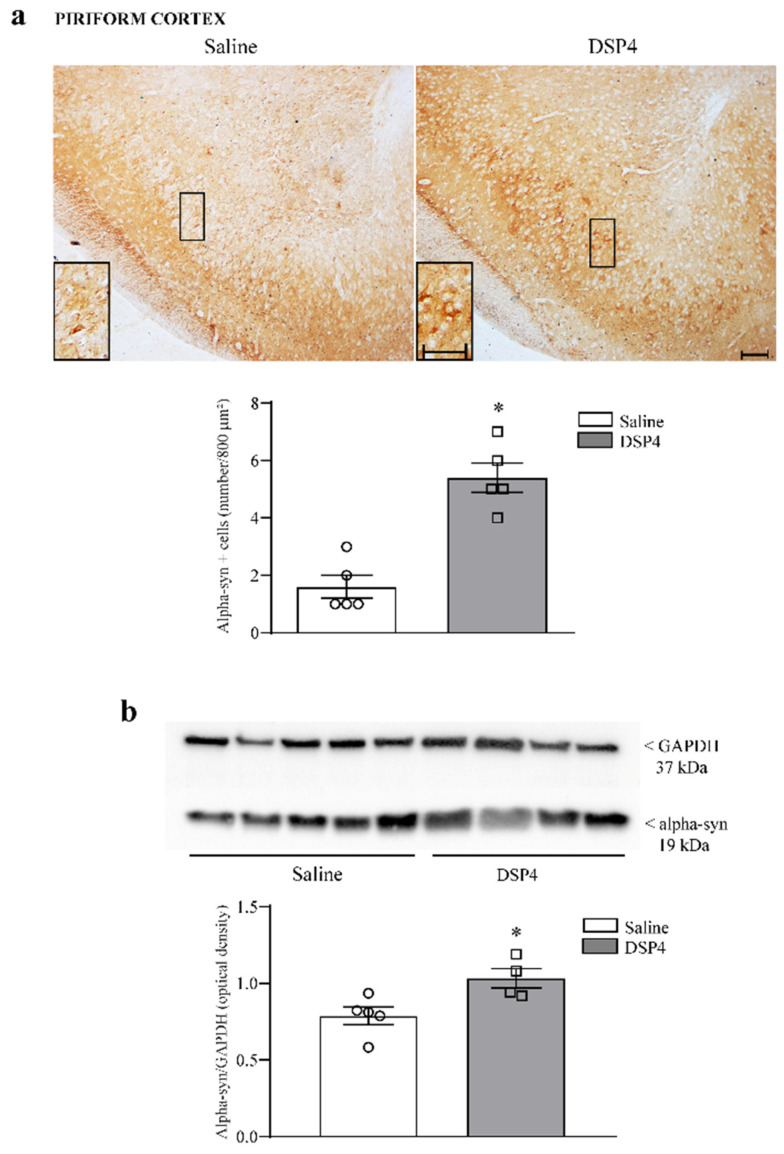
DSP-4 increases the number of alpha-syn-positive neurons and the expression of alpha-syn within the piriform cortex. In the upper part in Figure 7a, representative pictures show slices from the anterior piriform cortex stained for alpha-syn, and the density of alpha-syn-positive cells is reported as the number of cells per 800 μm^2^. An increase in alpha-syn-positive cells following DSP4 is mostly evident in the deep layer of the piriform cortex. This is shown within the insert reporting such a detailed layer of this cortical area at higher magnification. The number of alpha-syn-stained cells is reported in the graph were counts of saline-injected mice are compared with DSP4-treated mice. Data are expressed as the mean ± S.E.M. In (**b**), immunoblotting for alpha-syn protein is reported from the whole piriform cortex. The increase in this protein is slighter compared with the increase in alpha-syn-expressing cells reported in (**a**). In (**a**,**b**), the effects obtained in the group administered saline (N = 5) and DSP4 (N = 4) were compared using one-way ANOVA with Sheffè’s posthoc test. The null hypothesis was rejected for *p* ≤ 0.05. * *p* ≤ 0.05 compared with saline. Scale bar = 100 μm; 20 μm (insert). Circles and squares represent values referred to each single saline- or DSP4—injected mouse, respectively.

In the piriform cortex, the pre-administration of DSP4 elevates the number of alpha-syn positive cells (Figure 7a), which occurs along with an increase in alpha-syn protein levels assessed at immunoblotting (Figure 7b).

**Figure 8:**
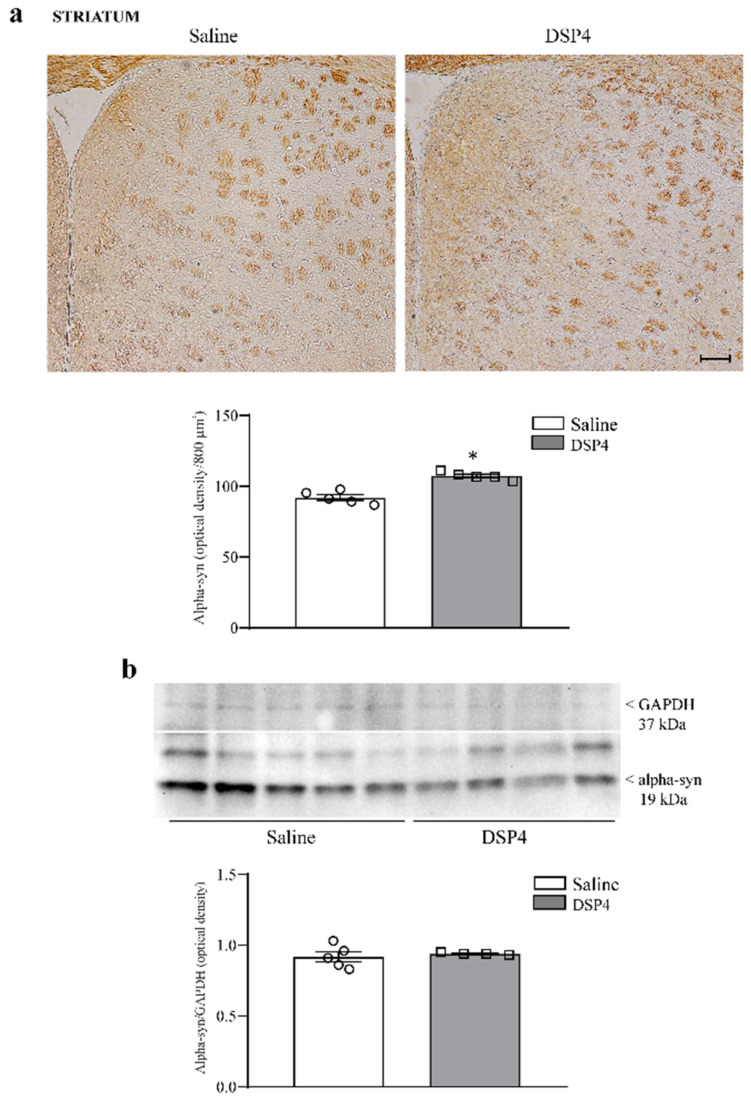
DSP4 slightly affects the amount of alpha-syn within the dorsal striatum. As reported in representative pictures placed in the upper part in Figure 8a, in slices from the dorsal striatum stained for alpha-syn, tissue staining density does not allow us to distinguish single cells. Therefore, it shows the amount of alpha-syn-stained tissue within specific spots from saline-injected mice and DSP-4-injected mice. Relative densitometry is expressed by optical tissue density compared with corpus callosum (as shown in Supplementary Figure S2). Even in this case, stained areas where optical density was calculated correspond to rectangles of 800 μm^2^, and they were chosen randomly within striatal tissue. Random sampling was needed since the staining was patchy, providing alternate pale and dark areas. The amount of alpha-syn staining slightly increases in mice administered DSP4, as reported in the graph, where counts in saline-injected mice are compared with DSP4-treated mice. Data are expressed as the mean ± S.E.M. In (**b**), immunoblotting for alpha-syn protein within the dorsal striatum is reported, with no significant effects induced by DSP4. In (**a**,**b**), the effects obtained in the group administered saline (N = 5) and DSP4 (N = 4) were compared using one-way ANOVA with Sheffè’s posthoc test. The null hypothesis was rejected for *p* ≤ 0.05. * *p* ≤ 0.05 compared with saline. Scale bar = 100 μm. Circles and squares represent values referred to each single saline- or DSP4—injected mouse, respectively.

Within the dorsal striatum of DSP4-administered mice, the amount of staining density for alpha-syn is comparable with the controls, although it appears to be slightly increased within the dorsal striatum following DSP4 (Figure 8a). No difference is detected concerning alpha-syn immunoblotting between the striatum from the controls and the striatum from DSP4-administered mice (Figure 8b).

### 2.4. DSP4 Elevates p-Tau within Limbic Regions

**Figure 9:**
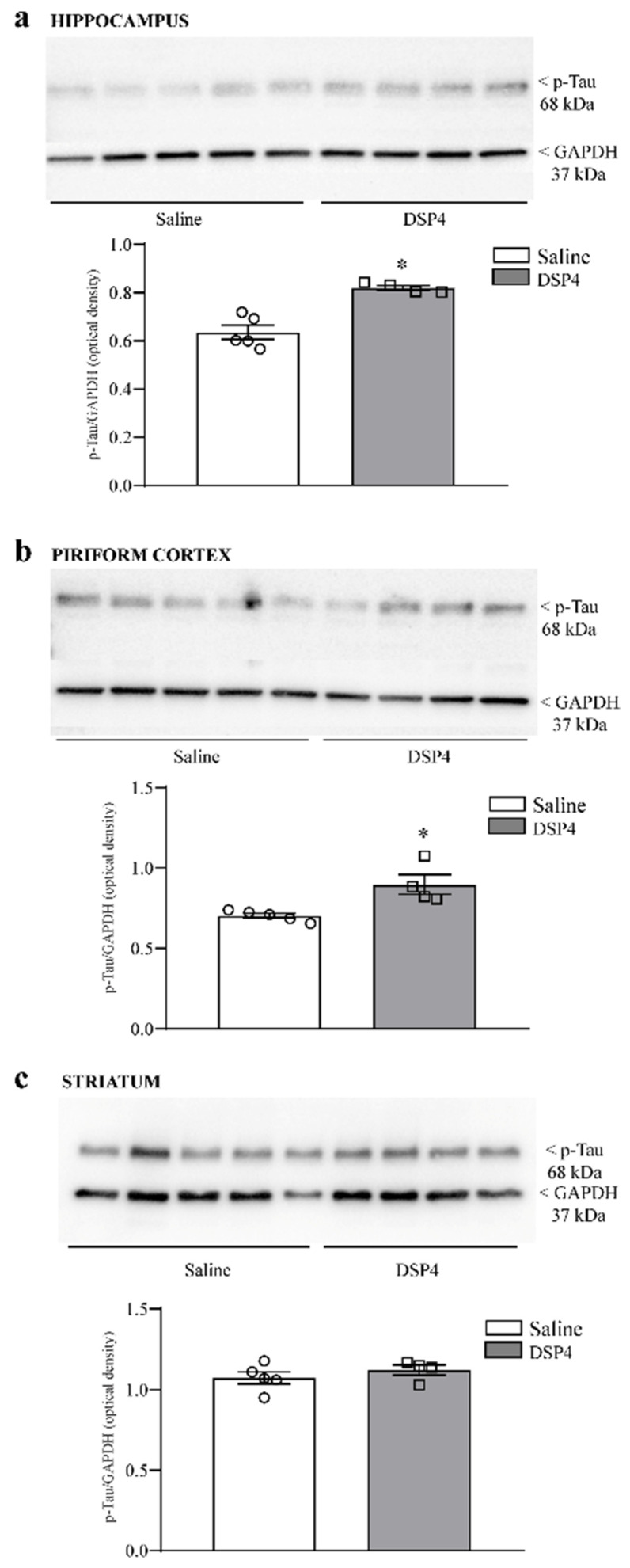
DSP4 increases p-Tau within limbic regions but not the dorsal striatum. Immunoblots were obtained from the hippocampus (**a**); piriform cortex (**b**); dorsal striatum (**c**); and saline- and DSP-4-injected mice. As reported in the graphs, semi-quantitative densitometry indicates an increase in p-Tau levels within limbic regions, such as the hippocampus (**a**) and piriform cortex (**b**), while no significant effect is detectable within the dorsal striatum (**c**). The effects obtained in the group administered saline (N = 5) and DSP4 (N = 4) were compared using one-way ANOVA with Sheffè’s posthoc test. The null hypothesis was rejected for *p* ≤ 0.05. * *p* ≤ 0.05 compared with saline. Circles and squares represent values referred to each single saline- or DSP4—injected mouse, respectively.

Similarly to alpha-syn, p-Tau protein was increased in the immunoblotting from the hippocampus (Figure 9a) and piriform cortex (Figure 9b) from DSP4-treated mice, while no significant effect was detected when immunoblotting the striatum from mice administered DSP4 compared with the controls (Figure 9c).

### 2.5. DSP4 Induces a Loss of p62 within Limbic Regions and the Dorsal Striatum

**Figure 10:**
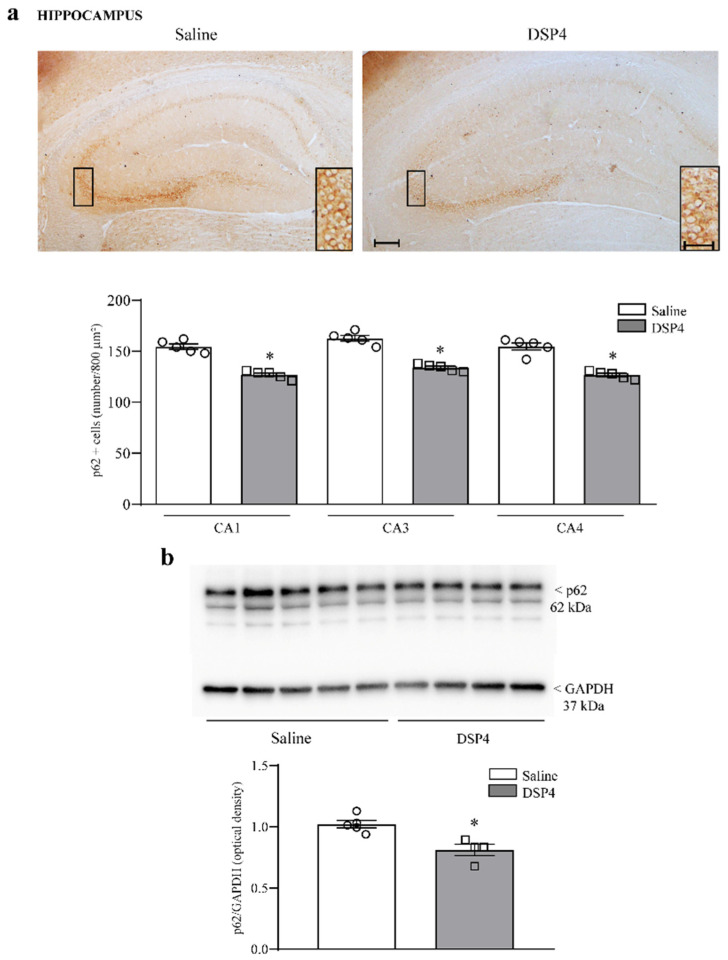
DSP4 decreases the number of p62-positive neurons and the expression of p62 protein within the hippocampus. In hippocampal slices stained for p62, the density of p62-positive cells was expressed as the number of cells per 800 μm^2^. These cells are evident in the upper part in Figure 10a, showing representative pictures, where the loss of p62-positive cells occurs in all sub-fields of *cornu ammonis* of the hippocampus, although the levels of p62 were more abundant within CA3 compared with other sub-fields. It is remarkable that no staining for p62 was detected within the dentate gyrus in the controls or in LC-NE-lesioned mice. The number of p62-stained cells is reported in the graph, where counts of saline-injected mice are compared with DSP4-treated mice. Data are expressed as the mean ± S.E.M. In (**b**), immunoblotting for p62 protein is reported from the whole hippocampus. In (**a**,**b**), the effects obtained in the group administered saline (N = 5) and DSP4 (N = 4) were compared using one-way ANOVA with Sheffè’s posthoc test. The null hypothesis was rejected for *p* ≤ 0.05. * *p* ≤ 0.05 compared with saline. Scale bar = 100 μm; 20 μm (insert). Circles and squares represent values referred to each single saline- or DSP4—injected mouse, respectively.

**Figure 11:**
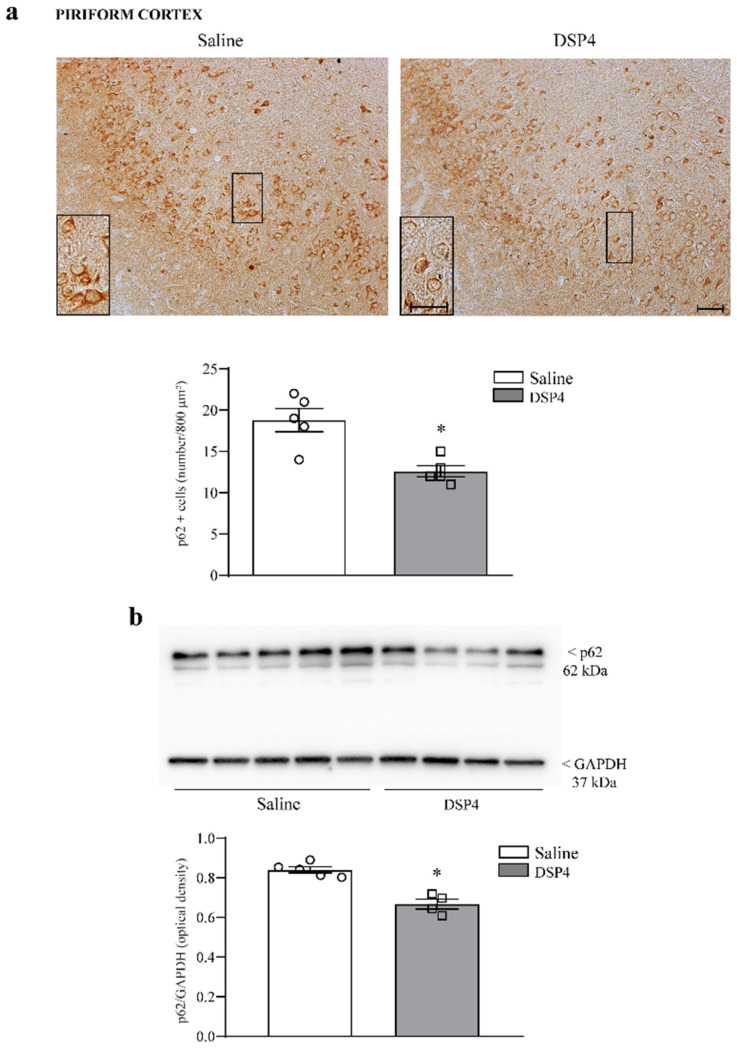
DSP4 decreases the number of p62-positive neurons and the the expression of p62 within the piriform cortex. In slices from the anterior piriform cortex stained for p62, the density of p62-positive cells is expressed by the number of stained cells per 800 μm^2^. These cells are evident in the upper part in Figure 11a, showing representative pictures, where the loss of p62-positive neurons in DSP4-treated mice occurs mostly in the deep layer of the piriform cortex, which is indicated in the insert at higher magnification. The number of p62-stained cells are reported in the graph, where counts of saline-injected mice are compared with DSP4-treated mice. Data are expressed as the mean ± S.E.M. In (**b**), immunoblotting for p62 protein is reported from the whole piriform cortex. In (**a**,**b**), the effects obtained in the group administered saline (N = 5) and DSP4 (N = 4) were compared using one-way ANOVA with Sheffè’s posthoc test. The null hypothesis was rejected for *p* ≤ 0.05. * *p* ≤ 0.05 compared with saline. Scale bar = 100 μm; 20 μm (insert). Circles and squares represent values referred to each single saline- or DSP4—injected mouse, respectively.

**Figure 12:**
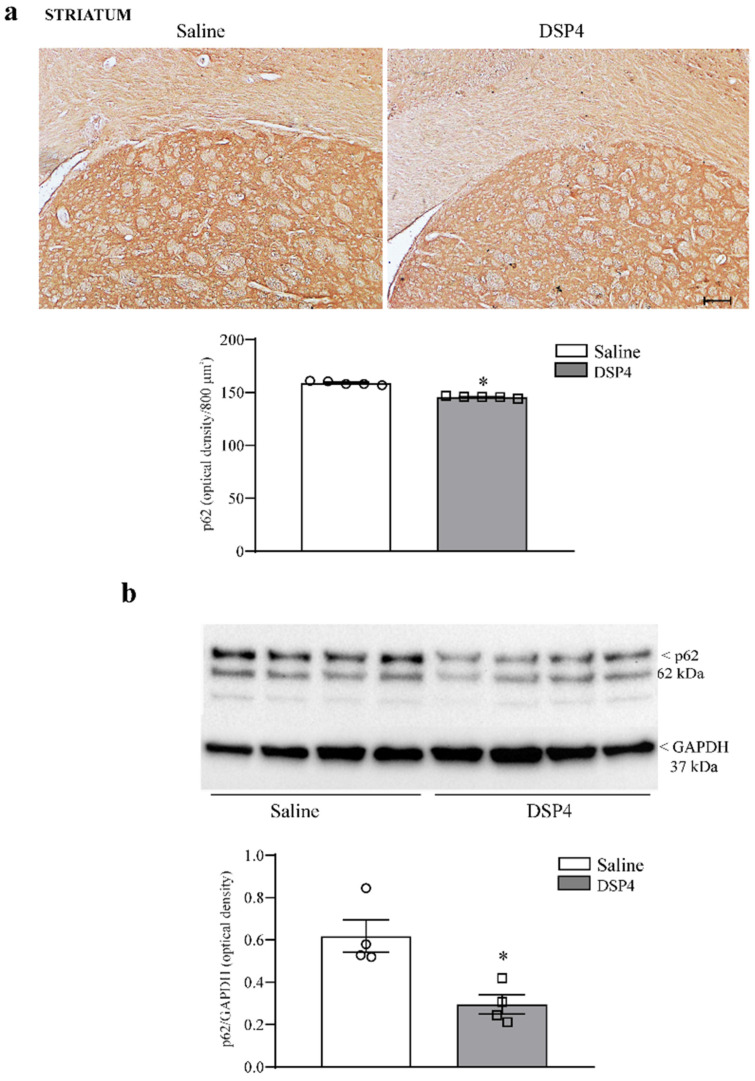
DSP4 does not modify the tissue density of p62 and the expression of p62 within the dorsal striatum. In (**a**), p62-stained slices from the dorsal striatum in the saline-injected controls or DSP4-injected mice did not allow us to detect any cell shape, as evidenced in representative pictures reported in the upper part in Figure 12a. Therefore, p62 immunostaining was reported as tissue density. In DSP4-treated mice, a slight decrease in optical density was observed compared with the controls, and it is reported in reference to corpus callosum, as shown in Supplementary Figure S2. These values are reported in the graph, where counts of saline-injected mice are compared with DSP4-treated mice. Data are expressed as the mean ± S.E.M. In (**b**), immunoblotting for p62 protein within the dorsal striatum is reported, which decreased in DSP4-treated mice compared with the saline-treated controls. In (**a**,**b**), effects obtained in the groups administered saline (N = 4) and DSP4 (N = 4) were compared using one-way ANOVA with Sheffè’s posthoc test. The null hypothesis was rejected for *p* ≤ 0.05. * *p* ≤ 0.05 compared with saline. Scale bar = 100 μm. Circles and squares represent values referred to each single saline- or DSP4—injected mouse, respectively.

The number of cells stained for the sequestosome protein p62 was moderately decreased in the hippocampus of mice administered DSP4 (Figure 10a). This occurs along with a slight decrease in the hippocampal levels of p62 protein assessed by immunoblotting (Figure 10b). Similarly, cell number, expressed as the number of p62-stained cells per 800 μm^2^, was reduced in the piriform cortex from DSP4-administered mice compared with the piriform cortex from the saline-injected controls (Figure 11a); such an effect was confirmed by a significant loss of p62 protein, as assessed by immunoblotting from DSP4-treated mice compared with the controls (Figure 11b). Within the striatum, immunostained tissue for p62 was slightly decreased following DSP4. At immunoblotting, the number of p62 protein was reduced to half of the control following DSP4 administration (Figure 12a and Figure 12b, respectively).

## 3. Discussion

The present data indicate that the i.p. administration of the selective NE neurotoxin DSP4 60 mg/Kg to C57 Black mice produces a specific NE loss, which is mostly abundant within forebrain limbic regions and is known to be critical in neurodegeneration, such as the hippocampus and piriform cortex. Serotonin levels, which decrease in other rodent species and albino mice, are similar to the controls without the need to administer a selective serotonin uptake inhibitor, which otherwise is mandatory in other rodent strains and species to prevent concomitant 5-HT toxicity [37,107]. Such a selective NE loss is shown to be selective since it depends on damage to LC-NE-arising axon terminals, as witnessed by a decrease in the amount of the pre-synaptic NE protein DBH and NE levels within LC target regions. This confirms recent validations about the established effects of such a neurotoxin [109], which turns out to be extremely specific for these NE axon terminals when used in C57 Black mice [110]. In each brain region, severe NE loss exceeds at large the loss of the specific presynaptic NE protein DBH. Such a discrepancy is likely to depend on the accuracy of the quantitative procedure assaying neurotransmitter levels from brain homogenates compared with the semi-quantitative densitometry of DBH immunoblotting. In addition, the loss of NE is more severe compared with the loss of DBH since a decrease in NE may also occur in axon terminals, where the storage mechanisms are impaired despite fair anatomical preservation. The wide spreading of LC-NE axons to pro-encephalon joined with their profuse branching suggests that the LC-NE pathway may act as a gating mechanism in modulating the threshold for neurodegeneration. Therefore, we questioned whether a loss of this subcortical pathway concomitantly affects the level and the staining of key proteins, playing either beneficial or detrimental effects in neurodegeneration. In fact, recent evidence suggests that an axon terminal damage of the LC-NE system is reminiscent of limbic degeneration, which occurs during early Alzheimer’s disease (AD) and PD and provides potential mechanisms underlying prodromal neuropsychiatric symptoms, which are grounded on the accumulation of deleterious proteins [111]. Based on such a strong background assessing severe damage to LC in the course of degenerative dementia and limbic involvement in PD, experimental studies in mammals and brain imaging techniques allow us to establish in vivo in humans a tight time course between LC degeneration and the occurrence of degenerative dementia [112,113,114,115,116]. The protective effects of LC-NE innervation are either worsened or improved by damage to LC neurons or a stimulation of NE receptors, respectively [110,117,118,119].

In line with this, damage to LC neurons was found to accelerate the deposition of p-Tau in combination with a high-sugar diet [120]. In this model, damage to LC accelerates and worsens neurodegeneration, which is typical of AD and dementia. These data follow up and update three decades of research connecting a loss of LC-NE innervation with the onset of degenerative disorders, such as PD and dementia [12]. This is why in the present study, key proteins involved in these disorders, such as alpha-syn and p-Tau, were assessed within critical limbic brain areas. In line with the experimental hypothesis linking damage of LC to the onset of specific degenerative disorders, we provide evidence that the amount of p-Tau is increased within the piriform cortex and hippocampus. Such an increase is significant, and it occurs within brain sites typically affected by degenerative dementia. In fact, the hippocampus features an increase in alpha-syn, and a similar effect is observed within the piriform cortex, mostly within the deep cortical layers. Remarkably, the piriform cortex is growingly recognized to be a primary site of neurodegeneration in dementia, and it is affected at the early stages of neurodegeneration when only a mild cognitive impairment is present at the clinical level [19,74,82,84,121,122,123]. In fact, the early degeneration of the piriform cortex is associated with mild cognitive impairment and odor loss, which is described in the pre-clinical stages of AD and PD [53,54,55,80,88,89,90]. In the present manuscript, the piriform cortex was recruited by alpha-syn and p-Tau accumulation at 7 days following the administration of DSP4. A loss of NE within the piriform cortex may lead to the onset of neurodegeneration acting at the pre-clinical level [4,70,73,74]. The primary physiological effect of the piriform cortex consists of odor recognition, association, and memory. This is in line with the occurrence of olfactory dysfunction in neurodegeneration, leading to the testing of olfactory activity during early AD for early diagnosis and monitoring subsequent steps in disease progression [74]. Similar findings are obtained in PD [124]. In strong connection with this, it is demonstrated that a loss of NE innervation is responsible for a loss of olfactory response in the piriform cortex, which is shown to be severely affected in the present study following a loss of LC-NE terminals. This is in line with the fact that the piriform cortex does not receive significant afferents from the thalamus, and the role of thalamic inputs is shifted to monoamine systems, mostly NE. Thus, LC-NE innervation of the piriform cortex may represent a key regulator of cortical excitability and plasticity [123,125]. In fact, the loss of LC-NE innervation generates a suppression of activity-induced *c-fos*-immunostaining and brain metabolism, which is remarkable within the piriform cortex [126]. This lends substance to a loss of acquired avoidance [127] and a loss of plasticity [128] following NE denervation of the piriform cortex. Such a pivotal role of NE in the piriform cortex may explain the suggestive findings obtained here concerning the increased amount of alpha-syn and p-Tau, joined with a severe loss of the neuroprotective chaperonin HSP70i and a decrease in the autophagy-related shuttle p62 following NE denervation. The novel findings reported in this manuscript add novel evidence to comprehend the relevance of NE integrity for the avoidance of neurodegeneration sustaining compensatory plasticity within the piriform cortex. This is consistent with the highest level of baseline NE levels in the piriform cortex compared with other brain areas analyzed in the present study and previous reports showing that NE levels in the piriform cortex surpass at large the amount of NE measured in other cortical regions, including the hippocampus. Consistently, the susceptibility of the piriform cortex to the loss of NE axons was the highest among the brain regions analyzed in the present study. Thus, the present data provide a strong association between the increase in deleterious proteins within brain areas critical for neurodegeneration and a loss of LC-NE innervation within the very same brain areas. Remarkably, this mirrors the early course of pathology and prodromal olfactory symptoms in the onset of degenerative disease. In fact, AD and PD patients experience deficiencies in odor detection, identification, and odor-related recognition memory [124,129]. A loss of NE innervation in the piriform cortex was shown to significantly impair the ability to receive and process olfactory information within this primary olfactory cortex [84].

The increase in alpha-syn and p-Tau described in the present study following damage to LC-NE axons is concomitant with a decrease in key proteins exerting a powerful protective effect in the very same neurodegenerative conditions. In fact, damage to LC-NE axons produces a severe loss of both HSP70i and p62 within the hippocampus and piriform cortex, while the effects within the dorsal striatum are slighter. The role of HSP70i is critical to provide neuroprotection. In fact, when excitotoxicity takes place in the limbic system, a compensatory increase in HSP70i is documented in the hippocampus and piriform cortex [130,131]. This is consistent with a powerful induction of the chaperonin HSP70i within allo-cortical brain areas in order to protect neurons [99]. The N-terminus of HSP70 is a key regulator of protein folding, which counteracts the pathophysiology of both PD and AD [132]. In fact, increasing the expression of HSP70i is known to mitigate neurodegeneration, while suppressing the expression of HSP70 promotes neurodegeneration in AD and PD [133]. Recently, a remarkable effect of HSP70 in promoting memory formation was reported [133]. Specifically, a recent manuscript by Akber (2021) [134] indicates that HSP70 is a key protein used to protect against deleterious effects induced by p-Tau, which enhances the dual effects of LC-NE axons in modulating the amount of these proteins within limbic areas, as evidenced in the present manuscript. The loss of p62 appears to have a significant value for the onset of neurodegeneration. In fact, the overexpression of p62 was shown to counteract the pathology of AD [135], while the suppression of p62 promotes alpha-syn accumulation and neurodegeneration [136]. The suppression of p62 observed in vivo following the destruction of LC-NE axons lends substance to the role of NE as an inducer of p62, as reported in vitro [137].

The present data are likely to be relevant for psychostimulant-induced limbic damage. In fact, methamphetamine produces alpha-syn pathology along with cognitive alterations [131,138].

## 4. Materials and Methods

### 4.1. Animals

For these experiments, we used 8-week-old C57Bl/6J male mice weighing 23–25 g (N = 20, Charles River, Calco, LC, Italy), which were either injected with saline (controls) or administered the norepinephrine neurotoxin DSP4. All animals were maintained under controlled environmental conditions (room temperature = 22 °C; humidity = 40%) on a 12 h light–dark cycle with food and water ad libitum. Animal care and experimentation were carried out according to institutional guidelines with the approval of the Italian Ministry of Health (authorization number #389/2023-PR).

### 4.2. Experimental Design

The experiments were aimed to detect the effects induced by the LC-NE neurotoxin DSP4 on the expression of specific proteins and the number of immunopositive neurons or immunostained tissue within the very same selected brain areas. In detail, HSP70i, p62, p-Tau, and alpha-syn were measured by Western blotting within limbic regions (hippocampus and piriform cortex) and within a non-limbic area (dorsal striatum). In parallel experiments, the number of neurons positive for HSP70i, p62, and alpha-syn were counted within the same brain regions following immunohistochemistry. The damage induced by DSP4 to NE-containing axons was assessed by immunoblotting the specific NE marker DBH and by the quantitative measurement of NE levels through high-performance liquid chromatography with electrochemical detection (HPLC-ED). These procedures allow us to infer the integrity of NE axon terminals within areas of interest. All procedures were carried out in mice sacrificed at 7 days. Mice were injected i.p. with either saline (equivalent to the controls) or they were administered DSP4 in a saline solution, such as DSP4 hydrochloride (Sigma-Aldrich, St. Louis, MO, USA), corresponding to 60 mg/Kg of DSP4 free base. The assessment of selective and severe damage induced by DSP4 to LC arising NE axons was needed to confirm, in the present experimental conditions, the selective neurotoxicity of DSP4, as previously reported in C57Bl/6J mice [110]. Damage to LC-NE-containing axons was massively induced within the piriform and hippocampal cortex, while the dorsal striatum was only slightly affected. This is consistent with the negligible number of NE axons in this brain area, partly coming from NE nuclei other than LC. Since damage to arising LC axons fosters degenerative phenomena involving limbic regions, such as the piriform and hippocampal cortex, this study aims to measure key proteins in neurodegeneration within these very same brain areas.

The choice of C57 Black/6J mice was based on a previous pioneer study showing that, although DSP4 is routinely used as a highly specific toxin tool to destroy NE axon terminals arising from the pontine nucleus LC in rodents, the crude evidence is quite different. In fact, following DSP4, the specificity and severity of monoamine depletion varies between rodent species and strains [107]. Thus, the pattern of monoamine depletion may diverge considerably from the early assumption that NE depletion occurs with a similar pattern in various rodent species and within various mouse strains. When DSP4 is administered to various rat strains, NE depletion is slighter compared with mice, and this is accompanied by a marked 5-HT loss. Even in albino Swiss–Webster mice, 5-HT is significantly reduced, and the amount of NE depletion may not be severe. When C57 Black mice are used, a remarkable and selective NE toxicity takes place, which is specific for LC target regions [110]. Since 5-HT levels are fully preserved following DSP4 in these mice, there is no need to co-administer a 5-HT re-uptake inhibitor concomitantly with DSP4, which is needed otherwise, when administering the neurotoxin to other mouse strains or rodent species. In monkeys, DSP4 produces a loss of NE levels in target regions combined with a loss of LC neurons; nonetheless, 5-HT levels in primates were not investigated [108].

Male mice (N = 10) were administered i.p. with DSP4 (Sigma Aldrich, MI, Italy, code: C-8417-250 mg) at a dose of 60 mg/Kg dissolved in 200 μL of saline to obtain a severe and pure loss of LC-NE axons. The control mice (N = 10) were administered 200 μL saline, which correspond to intact mice and naïve mice. Seven days after treatment, all mice were sacrificed under deep anesthesia (inhalation of isoflurane 2%), and brains were carefully removed from the skull to preserve the integrity of various areas, which were dissected for tissue sampling aimed at histochemistry, immunohistochemistry, monoamine assay, and Western blotting. In detail, in each group (saline or DSP4), N = 5 mice were analyzed for immunohistochemistry and histochemistry using both hemispheres, while N = 5 were used for immunoblotting and monoamine assay.

### 4.3. Tissue Sampling

In each mouse, the brain was placed into a Carnoy fixing solution composed of ethyl alcohol (60%), acetic acid (10%), and chloroform (30%). Twenty-four hours later, brains were placed into 70% ethanol to be embedded in wax (paraffin). The brains were cut at microtome (Leica Microsystem, RM2125, Milan, Italy) to obtain 10 μm thick coronal slices. Brain slices were collected starting from the frontal pole (bregma +3.08 mm according to the atlas of Paxinos and Franklin, 2004 [139]) and progressed caudally down to the bregma −1.90 mm, corresponding to the dorsal extent of the ventral hippocampal formation and including the thin layer of the *indusium griseum* (the real dorsal hippocampus placed above the corpus callosum).

In the present study, a special emphasis was placed on specific brain areas. In detail, the first set of slices included the levels of the prefrontal and piriform cortex. In addition to the hippocampus, which is the paradigm of limbic areas, the piriform cortex was collected based on recent findings, showing the relevant role of these allo-cortical sites as being primarily involved in degenerative dementia. These brain regions were punched for immunoblotting and sliced for immunohistochemistry. Slices analyzed were spaced 80 μm apart, and the piriform cortex extended from the bregma +3.20 mm to the bregma +1.70 mm. At this level, the rostral striatum was already present in its rostral pole, along with the piriform cortex. However, the striatal tissue was better analyzed more caudally, starting from the bregma +1.60 mm to the bregma −0.10 mm from serial slices 80 μm apart. A third set of slices was cut serially 80 μm apart to include the hippocampus, which was analyzed starting from the bregma −1.30 mm to the bregma −1.90 mm.

### 4.4. Histochemistry

For histochemical analysis, slices were de-waxed and processed for staining with hematoxylin–eosin. Specimens were placed in hematoxylin solution (Sigma-Aldrich) for 8 min. The slices were washed and plunged into eosin solution (Sigma-Aldrich) for 3 min. After repeated washing to remove excess dye, cells were dehydrated in increasing alcohol solutions, clarified in xylene, covered with mounting medium (Sigma-Aldrich), and finally observed under a Zeiss microscope (Axioimager M1, Zeiss, Oberkochen, Germany) equipped with a Nikon DS-Ri1 camera (Nikon, Tokyo, Japan).

Representative pictures of H&E staining were collected from the piriform cortex and hippocampus of both groups of mice. This procedure allows us to roughly appreciate the staining of cells present within a given brain area. In addition, this analysis was used as a preliminary observation to assess whether LC-NE denervation alters cell morphology.

### 4.5. Immunohistochemistry

Paraffin-embedded brain slices (10 μm) were used for immunoperoxidase.

These slices were stained with rabbit polyclonal anti-alpha-syn (Sigma Aldrich, dilution 1:100), monoclonal mouse anti-HSP70i (R&D Systems, Minneapolis, MN, USA, dilution 1:100), and recombinant rabbit anti-p62 (Abcam, Cambridge, UK dilution 1:100). Slices were treated with normal serum for 1 h (10% in TBS). Then, they were incubated overnight (4 °C) with primary antibody anti-alpha-syn, anti-HSP70i, and anti-p62 and then for 1 h with secondary biotinylated-coupled anti-mouse or anti-rabbit IgG (Vector Labs, Newark, CA, USA), (for detail see Table 1). Control staining was performed without primary antibodies.

Immunostaining was quantified by measuring the number of immunopositive cells (or the density of immunostained tissue when single cells could not be detected) from five different levels in each mouse (spaced 80 µm apart for the piriform cortex, hippocampus, and striatum, and N = 5 for each experimental group).

Cell count was carried out by light microscopy at 20× magnification; the number of stained cells within each area detectable after each specific treatment was counted, and the density of stained cells was expressed as the mean of cell number ± SEM per 800 μm^2^.

### 4.6. SDS Page Immunoblotting

The right hemisphere from N = 5 mice was micro-dissected to obtain samples for immunoblotting, while the left hemisphere was processed for HPLC-ED analysis. Dissections included the piriform cortex (from bregma level +1.30 mm to +0.98 mm), hippocampus (from bregma level −1.06 mm to −1.90 mm), and striatum (from bregma level +0.70 mm to +0.30 mm). The samples were homogenized at 4 °C in an ice-cold lysis buffer with phosphatase and a protease inhibitor. One μL of homogenates was used for protein determinations (Bradford procedure). Proteins (20 µg) were separated on sodium dodecyl sulfate gels (polyacrylamide PVDF precast gel 4–20% gradients) and transferred on transblot-turbo (Biorad, Hercules, CA, USA) for 7 min and mixed molecular weight. Filters were blocked for 2 h in Tween-20 Tris-buffered saline (TTBS, 100 mM TrisHCl, 0.9% NaCl, 1% Tween 20, pH 7.4) containing 5% non-fat dry milk (Biorad). Blots were incubated overnight at 4 °C with the following primary antibodies: mouse monoclonal anti-HSP70i (1:1000; R&D Systems), rabbit polyclonal anti-alpha-syn (1:1000; Sigma Aldrich), rabbit recombinant anti-p62 (1:1000; Abcam), mouse monoclonal anti-DBH (1:1000; Merck Millipore), and rabbit polyclonal anti-p-Tau (1:1000, sigma Aldrich). As a housekeeping protein, we used beta-actin (HSP70i) or GAPDH (other proteins), depending on the molecular weight and the migration of the specific protein under analysis. Blots were incubated with primary mouse monoclonal anti-beta-actin or GAPDH antibody (1:25,000, Sigma Aldrich, and 1:1000, Santa Cruz Biotechnology, Dallas, TX, USA, respectively) for 1 h at room temperature. The filter was washed 3 times with TTBS buffer and then incubated for 1 h with secondary peroxidase-conjugated antibodies (anti-mouse, 1:3000; Calbiochem, Milan, Italy). Immunostaining was revealed by enhanced chemiluminescence (GE Healthcare, Milan, Italy).

Bands of housekeeping (either beta-actin or GAPDH) and each specific protein (HSP70, alpha-syn, p-Tau, DBH, p62) were obtained by staining the very same membrane.

Densitometric analysis was performed with IMAGEJ software (NIH, USA, Version 1.8.0_172). Data were expressed as the mean ± S.E.M.

### 4.7. HPLC Assay

Samples from each brain area and each experimental group (N = 5 per experimental group, per each area) were carefully dissected from the brain. In detail, the right striatum was dissected through the lateral ventricle, while the right hippocampus was easily dissected by removing the subcortical region adjacent to the lateral ventricle caudally to the striatum. The right piriform cortex was removed from the ventral cortical surface in the corresponding temporal region. Each sample was placed within an Eppendorf containing 0.3 mL of ice-cold 0.1 M perchloric acid (Sigma-Aldrich). After sonication, an aliquot of the homogenate (50 µL) was assayed for protein [140]. After centrifugation at 8000× *g* for 10 min, 20 µL of the clear supernatant was stored immediately at 80 °C until use or directly injected into an HPLC system, where NE and 5-HT were analyzed as previously described using a highly lipophilic mobile phase as previously reported [110], running through a reversed-phase column (250 × 4.5 mm, C18) and two coulometric electrochemical detectors [110]. The reducing electrode was used for the quantitative analysis. The mobile phase consisted of a citrate-phosphate buffer (0.04 M citric acid, 0.06 M Na_2_HPO_4_·2H_2_O, Sigma-Aldrich) containing 0.1 mM EDTA (Sigma-Aldrich), 0.6 mM 1-heptanesulphonic acid sodium salt (Sigma-Aldrich), and 10% methanol (Sigma-Aldrich).

### 4.8. Open-Field Test

Locomotor activity was monitored in an open-field apparatus consisting of plexiglass test boxes (42 × 42 × 21 cm) associated with an activity monitor equipped with an infrared photoelectric beam interruption sensor (Open Field Activity System Hardware, Med Asso-ciates, Inc., St. Albans, VT, USA). Seven days after treatment with saline or DSP4 (60 mg/kg, i.p.), mice were individually placed in the text box, locomotor activity was recorded for 60 min time intervals, and data were expressed as ambulatory distance recorded during 12 time fragments lasting 5 min each. During these 5 min, the total locomotor distance was calculated per each mouse, and the results are reported as the mean ± S.E.M. of five mice per each group. Comparisons between groups were carried out using ANOVA with Sheffè’s posthoc analysis. The null hypothesis was rejected for *p* ≤ 0.05.

### 4.9. Statistical Analyses

Slices analyzed were spaced 80 μm apart. The piriform cortex extended from the bregma +3.20 mm to the bregma +1.70 mm. At this level, the rostral striatum was already present in its rostral pole, along with the piriform cortex. However, striatal tissue was better analyzed more caudally starting from the bregma +1.60 mm to the bregma −0.10 mm from serial slices 80 μm apart. A third set of slices was cut serially 80 μm apart to include the hippocampus, which was analyzed starting from the bregma −1.30 mm to the bregma −1.90 mm. Five slices from each of the 5 levels from each brain region were considered from each mouse (N = 5). This leads to a total of 125 areas for each brain region for each antigen from each group. Random sampling was needed, considering the variability of antigen expression within a specific brain area. This was mostly evident where single cells were not evident and/or a patchy staining was detected, such as in the case of alpha-syn within the hippocampus and striatum or p62 within the striatum.

When the densitometry of immunostained tissue was assessed (alpha-syn within striatum and hippocampus or p62 within striatum), the optical densitometry was expressed as a ratio between semi-quantitative density within the specific area under analysis and the density of the corpus callosum. Thus, tissue densitometry within wide areas of the striatum and hippocampus was carried out by sampling randomly five rectangles each, one measuring an area of 800 μm^2^ (20 μm × 40 μm). The values of semi-quantitative densitometry were obtained using Image J software (NIH, USA, Version 1.8.0_172). Ratios of densitometry expressing tissue immunostaining were reported as the mean ± S.E.M. Inferential statistics were used to compare groups using one-way ANOVA. The null hypothesis H_0_ was rejected for *p* ≤ 0.05.

When the count of cell density was carried out, 5 areas from each level from each brain region were considered from each mouse. This led again to count a total of 125 random areas from each brain region from each antigen in each group (N = 5). Thus, the number of cells was calculated by counting immunopositive cells within rectangles of 800 μm^2^ (20 μm × 40 μm), which were reported as the mean ± S.E.M. for each area. Inferential statistics to compare groups was carried out using Student’s *t*-test (H_0_, the null hypothesis, was rejected when *p* ≤ 0.05).

For Western blotting experiments, values of optical density were presented as the mean ± S.E.M of the ratios between each protein under analysis and the housekeeping protein. Comparisons between the controls and DSP4 were carried out using one-way ANOVA. The null hypothesis H_0_ was rejected for *p* ≤ 0.05.

HPLC analysis was carried out by preliminary assaying NE and 5-HT to draw standard curves. In detail, known amounts of NE and 5-HT (Sigma-Aldrich) were dissolved in perchloric acid (0.1 N) containing a constant amount (10 pg/μL) of the internal standard (dihydroxybenzylamine, DBA; Sigma-Aldrich), and it was calculated using the regression analysis of ratios between peak areas (NE or 5-HT area/DBA area) for various NE and 5-HT concentrations recorded at the reducing electrode. Values are given as the mean ± S.E.M. of values obtained in each experimental group. Quantitative amounts of NE and 5-HT were obtained by regression analysis, and they were expressed as ng/mg of protein. Comparisons between groups were carried out using one-way ANOVA. The null hypothesis H_0_ was rejected for *p* ≤ 0.05.

## 5. Conclusions

The marked effects of LC-NE denervation on the expression of proteins, which are key in the relevant steps of neurodegeneration and neuroplasticity within the limbic system, represent a further piece of evidence provided by the present study in the frame of the unique role of the pontine nucleus LC to sustain the integrity and promote the recovery of brain regions relevant for a number of degenerative disorders. Based on the present findings, the degeneration of LC is expected to suppress the expression of proteins, such as HSP70 and p62, which are pivotal in sustaining neuronal integrity and counteracting neuronal damage. In turn, this is seminal in fostering the onset and progression of degeneration and dementia. This is directly related to the demonstration provided here that LC-NE damage increases the expression of p-Tau and alpha-syn, which are known to accumulate in the course of neurodegeneration. This is consistent with imaging in patients affected by AD [141]. Limbic site specificity, which emerges, is consistent with the key role of brain NE to promote plasticity within these areas. A special emphasis deserves the unique efficacy of LC-NE loss in altering such a protein expression within the neurons of the piriform cortex. In fact, this region possesses the highest amount of NE among cortical areas. Remarkably, early NE denervation in the piriform cortex occurs in mild cognitive impairment and early-stage dementia. Due to a mixed pathology in degenerative dementia, where primary neuronal damage is often concomitant with vascular alterations, the outcome of damage to LC neurons should also be tested in a consistent model of brain ischemia. In addition, the present findings implement the chain of events bridging damage to LC neurons to altered plasticity within the limbic system with an emphasis on the piriform cortex, which was often missed in classic studies of degenerative disorders, but it is presently emerging as a fundamental area in recent neuropathological investigations. In a plain representative H&E histochemistry of the hippocampus and the piriform cortex (Supplementary Figure S3 and Supplementary Figure S4, respectively), the main difference between DSP4 and the saline group appears as a pale staining concerning hematoxylin and eosin, while the cell number is not modified. These images serve to foster further research studies aimed at identifying subcellular changes induced by LC-NE damage within limbic target regions. Both figures report two representative pictures from an LC-NE-damaged mouse and a control mouse. The difference is stunning and deserves in-depth and prompt investigations. This calls for in-depth and extensive behavioral evaluations. In the present manuscript, we included some behavioral data obtained by measuring open-field activity. In DSP4-treated mice, we detected a slight decrease in the open field, as reported in Supplementary Figure S5. These data confirm that was recently reported by Iannitelli et al. (2022) [111]. In fact, as reported in the graph (Supplementary Figure S5), mice administered DSP4 7 days after treatment showed decreased locomotor activity compared with the saline-injected controls. The open field was measured during 60 min time intervals, and it was expressed as measurements of ambulatory distance (cm) during time fragments of 5 min.

In the present study, there is a need to implement extensive investigations aimed to address (i) whether the effects in the inducible form of HSP70 are replicated for constitutive HSP70 and (ii) which behavioral test may be specifically altered by an NE-depleted brain. In fact, we report some preliminary observations and call for an in-depth and extensive behavioral evaluation spanning the whole domain of LC effects in behavior. (iii) Intracellular alterations induced by LC-NE denervation of the limbic system that may foster neurodegeneration should also be studied. In fact, beta-2 signaling was recently found to be critically related to the protective effects of NE within neuronal cells by promoting autophagy [119]. Finally, the subcellular organelles and clearing pathways critically involved in protein accumulation during degenerative disorders deserve specific investigation. This is related to persistent aberrant plasticity, which may be triggered by a loss of the LC-NE system and should be investigated by molecular biology. The LC-NE-depleted brain appears grossly different, as shown in Supplementary Figures S3 and S4, which calls for deciphering which pathways in cell pathology are mostly engaged. This is likely to mimic the early steps of main degenerative disorders extending to the cytopathology of the addicted brain.

## Figures and Tables

**Table 1:** Primary and secondary antibodies used in this study.

Antibody	Provider	Catalog Number	RRID	Concentration
Monoclonal mouse anti-HSP70 (l.m.; w.b.)	R&D Systems	Cod. MAB-1663	AB 2119388	1:1000
Polyclonal anti-p-Tau (w.b.)	Sigma Aldrich	Cod. P-7444	*	1:1000
Horse anti-Mouse IgG antibody (H + L), biotinylated (l.m.)	Vector Labs	BA-2000-1.5	AB_2313581	1:200
Mouse anti-beta-actin (w.b.)	Sigma Aldrich	A1978	AB_476692	1:25,000
Mouse anti-GAPDH (w.b.)	Santa Cruz Biotechnology	SC32233	AB_627679	1:1000
Mouse monoclonal anti-dopamine-beta-hydroxylase (w.b.)	Millipore	MAB308	AB_2245740	1:1000
Rabbit polyclonal anti-alpha-synuclein (l.m.; w.b.)	Sigma Aldrich	SAB4502828	AB_10746104	1:1000 w.b. 1:100 l.m.
Horse anti-Rabbit IgG antibody (H + L), Biotinylated (l.m.)	Vector Labs	BA-1100	AB_2336201	1:200
Recombinant rabbit anti-SQSTM1/p62 (l.m.; w.b.)	Abcam	AB109012	AB_2810880	1:1000 w.b. 1:100 l.m.
Goat anti-rabbit (w.b.)	Millipore	401-393	AB_437797	1:3000
Goat anti-mouse (w.b.)	Millipore	401-215	AB_10682749	1:3000

l.m. = light microscopy; w.b. = Western blotting. * RRID is not available in the online database; it can be provided upon request.

## Data Availability

The data that support the findings of this study are available from the corresponding author upon reasonable request. The raw data supporting the conclusions of this article will be made available by the authors on request.

## Supplementary Materials

The following supporting information can be downloaded at https://www.mdpi.com/article/10.3390/ijms25063159/s1.
