# Supplementary material for: Damage to the Locus Coeruleus Alters the Expression of Key Proteins in Limbic Neurodegeneration
Source: Int J Mol Sci. 2024 Mar 9;25(6):3159. doi: 10.3390/ijms25063159 (PMC10970344; doi:10.3390/ijms25063159)
Supplement: Supplementary file 1 [file ijms-25-03159-s001.zip › ijms-2868231-supplementary.pdf]

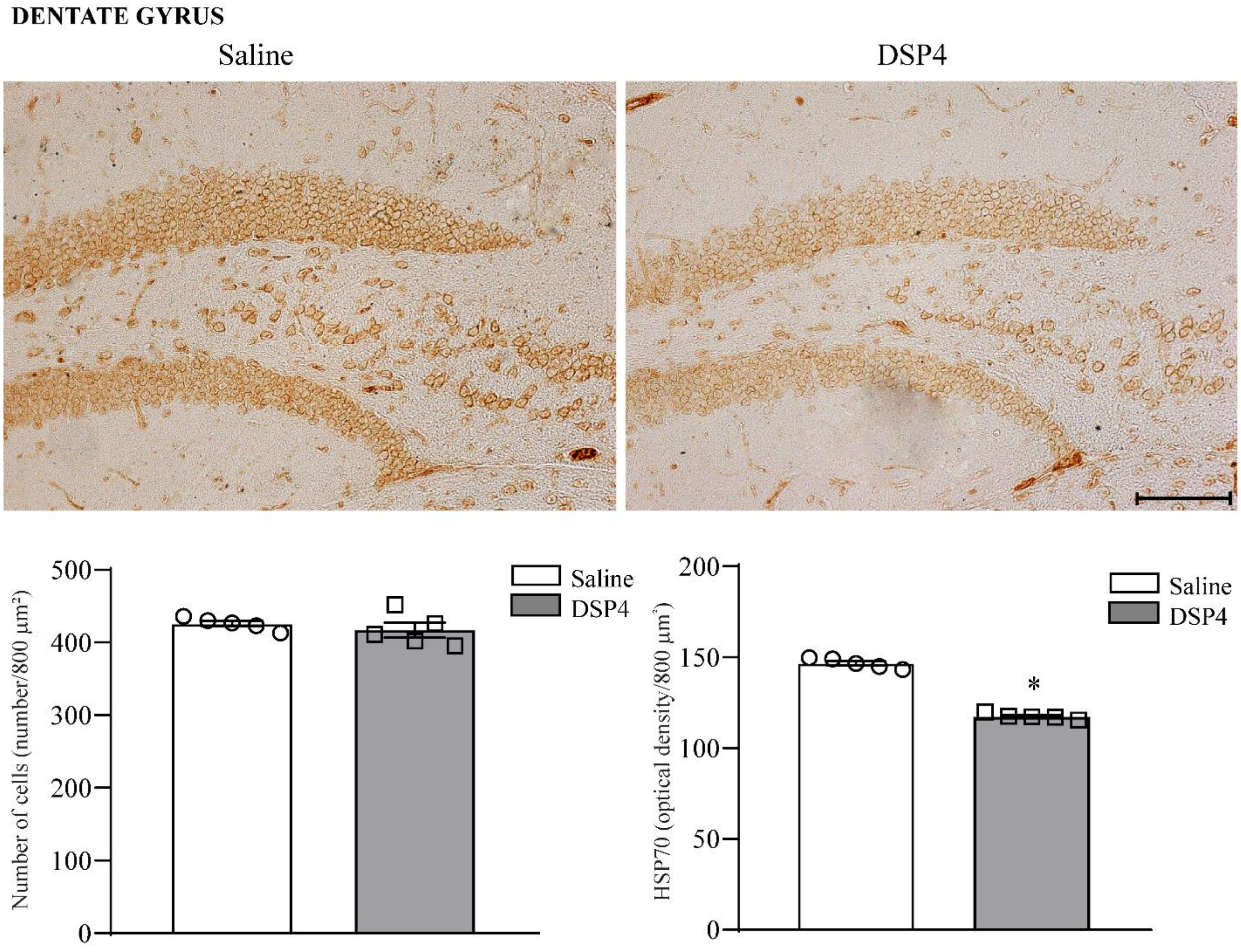

Supplementary Figure S1. Cell density and immunostained tissue densitometry within dentate gyrus.

In the upper line of the panel representative pictures show that DSP4 does not modify the amounts of HSP70i-positive neurons within dentate gyrus compared with controls. Nonetheless, when the density of immunostaining was calculated DSP4 decreased the amount of staining per cell which produced a pale staining within dentate granule cells DSP-4 administered mice compared with controls. In the second line of the panel the graph on the left indicates no variation in the number of cells per 800 μm^2, while in the graph on the right the staining density indicates that DSP4 decreases significantly the staining of granule cells. The amounts of HSP70i-stained cells is reported in the left graph were counts of saline-injected mice are compared with DSP4-treated mice. The amount of cellular staining for HSP70i is reported in the graph on the right. In this way a loss of the protein HSP70i appears following DSP4 although the number of positive cells is similar to controls. Data are expressed as the mean±S.E.M. in each group administered either saline (N=5) or DSP4 (N=5) data were compared by using one-way ANOVA with Sheffè's *post-hoc* analysis. Null hypothesis was rejected for $p \leq 0.05$. *$p \leq 0.05$ compared with saline. Scale bar=100μm.

CORPUS CALLOSUM

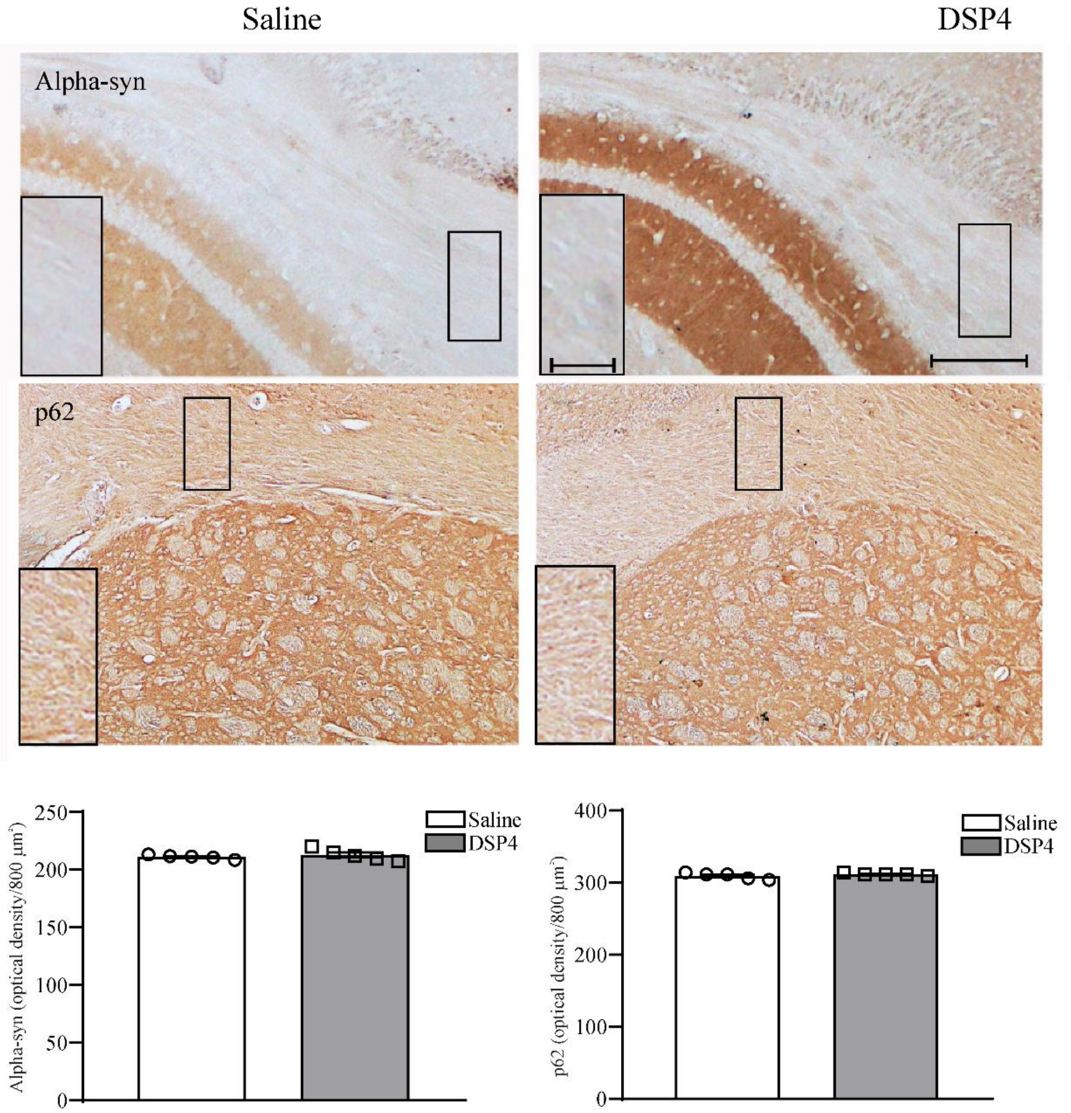

Supplementary Figure S2. Alpha-syn and p62 densitometry within correspondent level of corpus callosum.

Since antigen densitometry within corpus callosum served as a reference for tissue densitometry assessed within hippocampus for alpha-syn and within striatum for alpha-syn and p62, here representative images are reported showing the staining for alpha-syn and p62 within corpus callosum from controls and DSP4. The magnification is increased in each insert to focus on the density of corpus callosum for each staining. In the graphs semiquantitative tissue densitometry of corpus callosum is not modified in DSP4- compared with saline-injected mice neither for alpha-syn (left graph) nor for p62 (right graph). Data are expressed as the mean+S.E.M. in each group administered either saline (N=5) or DSP4 (N=5) data were compared by using one-way ANOVA with with Sheffè's *post-hoc* analysis. Null hypothesis was rejected for p≤0.05. *p≤0.05 compared with saline. Scale bar main figure=100µm; Scale bar insert=20µm.

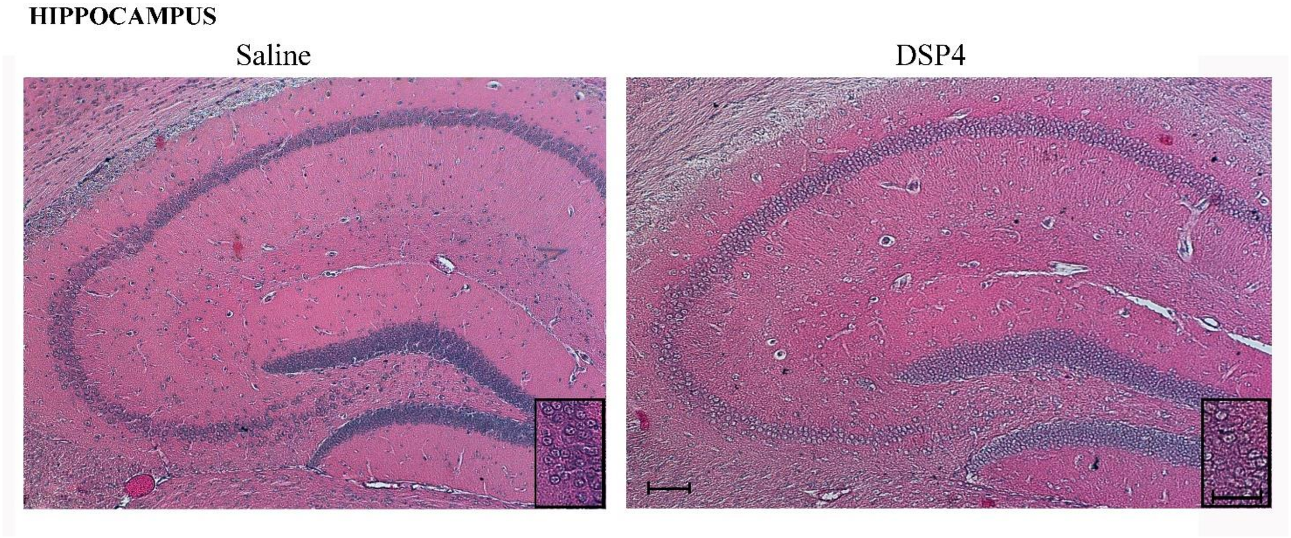

Supplementary Figure S3. Histochemistry with Hematoxylin & Eosin showing the hippocampus from a control mouse and a mouse administered DSP4. It is evident the pale staining of hippocampal cells following DSP4 administration compared with controls. Scale bar main figure=100μm; Scale bar insert=20μm.

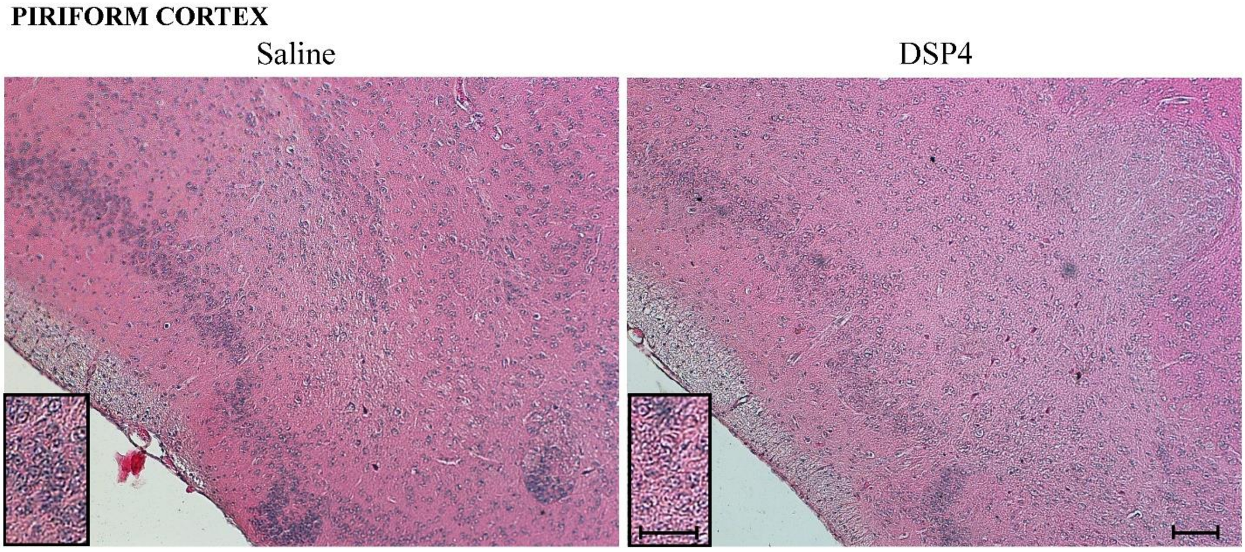

Supplementary Figure S4. Histochemistry with Hematoxylin & Eosin showing the piriform cortex from a control mouse and a mouse administered DSP4. It is evident the pale staining of allo-cortical cells following DSP4 administration compared with controls. Scale bar main figure=100μm; Scale bar insert=20μm.

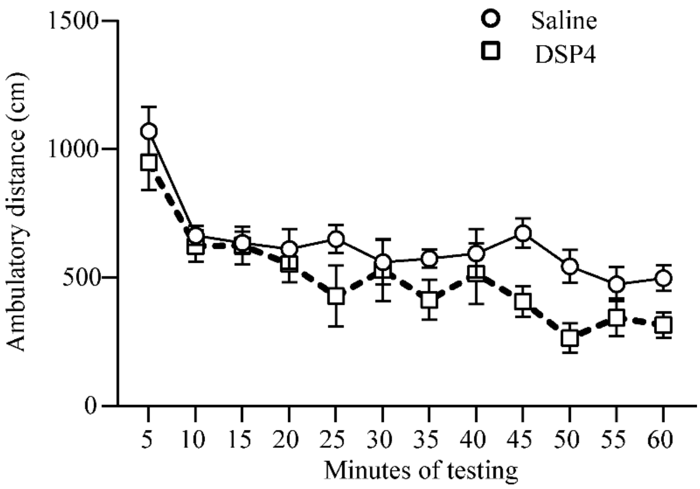

Supplementary Figure S5. Open field activity. Mice administered DSP4 (N=5) possess, at 7 days after treatment, decreased locomotor activity compared with control mice injected with saline (N=5). Each open field section was lasting for 60 min time intervals and it was carried out during light time (11:00-12:00 A.M.). Locomotor activity was expressed as ambulatory distance covered by each mouse during time fragments of 5 min each. Results are reported as the mean+S.E.M. of five mice per group. Inferential statistics was calculated by using ANOVA with Sheffè's *post-hoc* analysis. Null hypothesis was rejected for $p \leq 0.05$.
